# Taxonomic evaluation of eleven species of *Microcyclops* Claus, 1893 (Copepoda, Cyclopoida) and description of *Microcyclops inarmatus* sp. n. from America

**DOI:** 10.3897/zookeys.603.7480

**Published:** 2016-07-06

**Authors:** Martha Angélica Gutiérrez-Aguirre, Adrián Cervantes-Martínez

**Affiliations:** 1 Universidad de Quintana Roo (UQROO), Unidad Cozumel, Av. Andrés Quintana Roo s/n, 77600, Cozumel, Quintana Roo México

**Keywords:** Diversity, Mexico, morphology, species richness

## Abstract

Description and meristic analysis of eleven species of *Microcyclops* recorded in America were performed based on the examination of type specimens and fresh material. Microscopic analysis of oral appendages, such as the shape and armature of the distal coxal endite of the maxilla, the ornamentation on the caudal surface of the antenna, and the intercoxal sclerites and armament of the inner basis of all swimming appendages, were characteristics that allowed the differentiation between species. Among these species, our study confirmed the synonymy of *Microcyclops diversus* Kiefer, 1935 with *Microcyclops ceibaensis* (Marsh, 1919). The results of our observations showed that *Microcyclops alius* (Kiefer, 1935) is a junior synonym of *Microcyclops dubitabilis* Kiefer, 1934; the latter being confirmed as a valid species. Also, it is proposed that the records of *Microcyclops rubellus* (Lilljeborg, 1901) and *Microcyclops varicans* (Sars, 1863) in America should be revised as there are serious doubts about their distribution in America. The analysis suggested that *Microcyclops anceps pauxensis* Herbst, 1962 is distinct from Microcyclops anceps var. minor Dussart, 1984 and that both are likely different from *Microcyclops anceps anceps* (Richard, 1897). Finally a full morphological description of adult females of *Microcyclops inarmatus* **sp. n.** is presented.

## Introduction

In America, 16 species and subspecies of *Microcyclops* Claus, 1893 have been described and recorded: *Microcyclops alius* (Kiefer, 1935), *Microcyclops anceps anceps* (Richard, 1897), *Microcyclops anceps pauxensis* Herbst, 1962, Microcyclops anceps var. minor Dussart, 1984, *Microcyclops ceibaensis* (Marsh, 1919), *Microcyclops dubitabilis* Kiefer, 1934, *Microcyclops echinatus* Fiers, Ghenne & Suárez-Morales, 2000, *Microcyclops elongatus* (Lowndes, 1934), *Microcyclops finitimus* Dussart, 1984, *Microcyclops furcatus* (Daday, 1905), *Microcyclops mediasetosus* Dussart & Frutos, 1985, *Microcyclops medius* Dussart & Frutos, 1985, *Microcyclops pumilis* Pennak & Ward, 1985, *Microcyclops rubellus* (Lilljeborg, 1901), *Microcyclops diversus* Kiefer, 1935, and *Microcyclops varicans* (Sars, 1863).

In her publication of an identification key for South American cyclopoids, Reid (1985) proposed that *Microcyclops diversus* is a probable synonym of *Microcyclops ceibaensis* (in 1986, this opinion was based on similarities in the fourth leg observed by the same author) and that Microcyclops anceps var. minor is a synonym of *Microcyclops anceps pauxensis*. Rocha (1998) proposed a set of morphological features that would be useful for distinguishing five species previously recorded in Brazil and suggested that *Microcyclops alius* is a possible synonym of *Microcyclops dubitabilis*. However, Reid (1990) had previously suggested that *Microcyclops dubitabilis* is a possible synonym of *Microcyclops varicans*.

Therefore, some questions on the taxonomic status of some species of *Microcyclops* in America remain unresolved. These taxonomic problems may be related to the lack of thorough and rigorous species descriptions. Rocha (1998), Mirabdullayev (1998, 2007), and Mirabdullayev and Urazova (2006) have documented morphological features that are useful for differentiating some species of the genus. For instance, they proposed the following morphological features as diagnostic: ornamentation of dorsal margins of prosomites, presence or absence of pores on second endopodite of first leg, ornamentation of the inner margin of basipodite of first leg, ornamentation of caudal ramus and caudal setae, relative lengths of caudal setae, proportions of second endopodite of fourth leg, and general ornamentation of fourth leg.

In Mexico, some species with uncertain taxonomic status have been recorded, including *Microcyclops ceibaensis*, *Microcyclops anceps*, and *Microcyclops dubitabilis* (Elías-Gutiérrez et al. 2008). In this paper, we propose a set of morphological features that are useful for distinguishing between these species, which have been documented by biological inventories of the country. These features include the mouth appendages, the ornamentation of intercoxal sclerites, and the inner margin of the basis of the first to fourth swimming legs.

## Methods

The morphological analysis was performed following current standards for the taxonomic study of cyclopoid copepods (see Williamson and Reid 2001).

**Material examined.** The evaluation included analyses of holotypes, paratypes, and museum specimens deposited in different collections: the Collection of Zooplankton of ECOSUR at Chetumal (ECO-CH-Z), the collection of Copepoda of the Muséum National d’Histoire Naturelle, Paris (MNHN), the Staatliches Museum für Naturkunde, Karlsruhe (SMNK) and the National Museum of Natural History, Smithsonian Institution, Washington, DC (USNM) (Table 2, as Suppl. material 1).

Terminology used for the armament of each appendage(s) follows Huys and Boxshall (1991):

A1
 Antennule 


A2
 Antenna 


BspA2
 Antennal basipodite 


Bsp
 Basipodite 


Enp1-Enpn
 First to “n” endopodal segment 


Exp1-Expn
 First to “n” exopodal segment 


P1, P2, P3, P4
 First, second, third, and fourth swimming legs 


P5
 Free segment of fifth leg 


Lateral, outermost terminal, outer median terminal, inner median terminal, innermost terminal, and dorsal caudal setae are coded as setae II, III, IV, V, VI, and VII, respectively.

The morphology of several species was examined using light microscopy: A1, A2, the mouthparts, the entire structure of all of the swimming legs, and other taxonomically relevant structures, such as the frontal or caudal ornamentation of BspA2, the ornamentation of the distal coxal endite of the maxilla, and the ornamentation of maxillular palp, were illustrated with the aid of a camera lucida.

Sources for the morphological data were the types, paratypes, and other museum specimens (Table 2, as Suppl. material 1), and original descriptions of eleven named species and two named subspecies recorded in America. Detailed descriptions based on the microscopic and morphometric analyses of the adult females of each species are presented.

## Results

### Descriptive section

Below those morphological structures which are shared by all the species examined herein are described.

**Figure 1.**
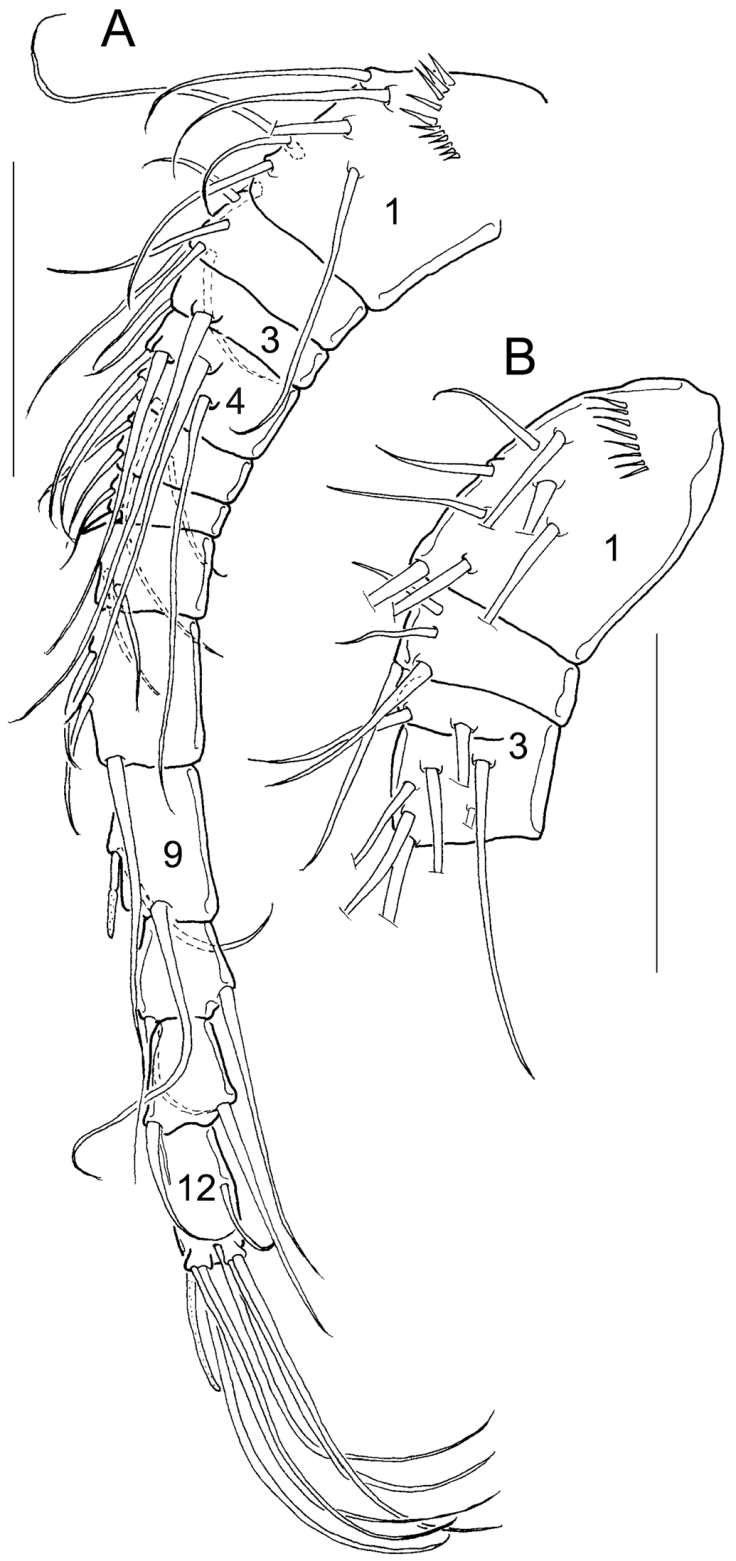
Morphology of antennules. **A** Antennule with 12 segments (*Microcyclops ceibaensis* from km 51-2) **B** Morphological variation in antennules with 11 segments (*Microcyclops dubitabilis* from km 51-2). Scale bars: 50 µm.

*Antennule* 11- or 12-segmented (Fig. 1). In the basic 12-segmented structure (Fig. 1A), each segment was armed with setae (s), spines (sp) or aesthetascs (ae) in the following order: (1) 8s; (2) 4s; (3) 2s; (4) 6s; (5) 3s; (6) 1s + 1sp; (7) 2s; (8) 3s; (9) 2s + 1ae; (10) 2s; (11) 2s + 1 ae; (12) 7s + 1 ae. In the case of the 11-segmented antennule the third and fourth segments are entirely or partially fused (Fig. 1B); then, the third segment bears 8s.

*Antenna* with coxa (without seta), Bsp (with 2 medial setae + one lateral seta representing Exp), and 3-segmented Enp (Fig. 3B). Labrum with strong teeth on distal rim and strong, distal hairs overhanging distal rim (Fig. 6D). Mandible with toothed gnathobase; the innermost teeth bi-toothed. Innermost margin of mandibular gnathobase with one spinulose seta, palp with two long and one short seta. No spinules next to mandibular palp (Fig. 6E).

*Praecoxal arthrite and palp of maxillule* naked; praecoxal arthrite with 3 chitinized distal claws, and one spinulose seta on caudal side. Inner margin with one biserially plumose seta plus six naked setae (Fig. 6F). Maxilla with praecoxa and coxa partially fused, praecoxal endite with two setae, coxa naked with proximal endite bearing one seta (Fig. 6L) and distal endite with two armed long setae (Fig. 6M). Claw-like basal endite armed, and Enp one- or two-segmented.

*Maxilliped* with syncoxa bearing 2 or 3 spiniform setae, Bsp with two setae; and Enp two-segmented, first segment with 1 seta, second segment with 3 setae (Fig. 6N).

*Armature formula* of P1−P4 as in Table 1, endopods and exopods two-segmented in all swimming legs. Urosome five-segmented, fifth pediger bearing one free segment with one apical seta (fifth leg), and one lateral seta inserted on pediger (Fig. 8C). Detailed description of the species is provided in the next section. The material examined for each species is provided in Table 2, as Suppl. material 1.

**Table 1.** Setation formula of the swimming legs in the *Microcyclops* species here examined (spine in Roman numerals, seta in Arabic numerals).

	Coxa	Basis	Exp	Enp
P1	0-1	1-I, or 1-0	I-1; III-5	0-1; 1-I-4
P2	0-1	1-0	I-1; IV-5	0-1; 1-I-5
P3	0-1	1-0	I-1; IV-5	0-1; 1-I-5
P4	0-1	1-0	I-0; III-5	0-1; 1-II-3

### Order: Cyclopoida Burmeister, 1835 Family: Cyclopidae Rafinesque, 1815 Subfamily Cyclopinae Rafinesque, 1815 Genus *Microcyclops* Claus, 1893

#### Microcyclops inarmatus sp. n.

Taxon classificationAnimaliaCyclopoidaCyclopidae

http://zoobank.org/687BDBC3-853D-437E-9310-4146F210094A

**Figure 2.**
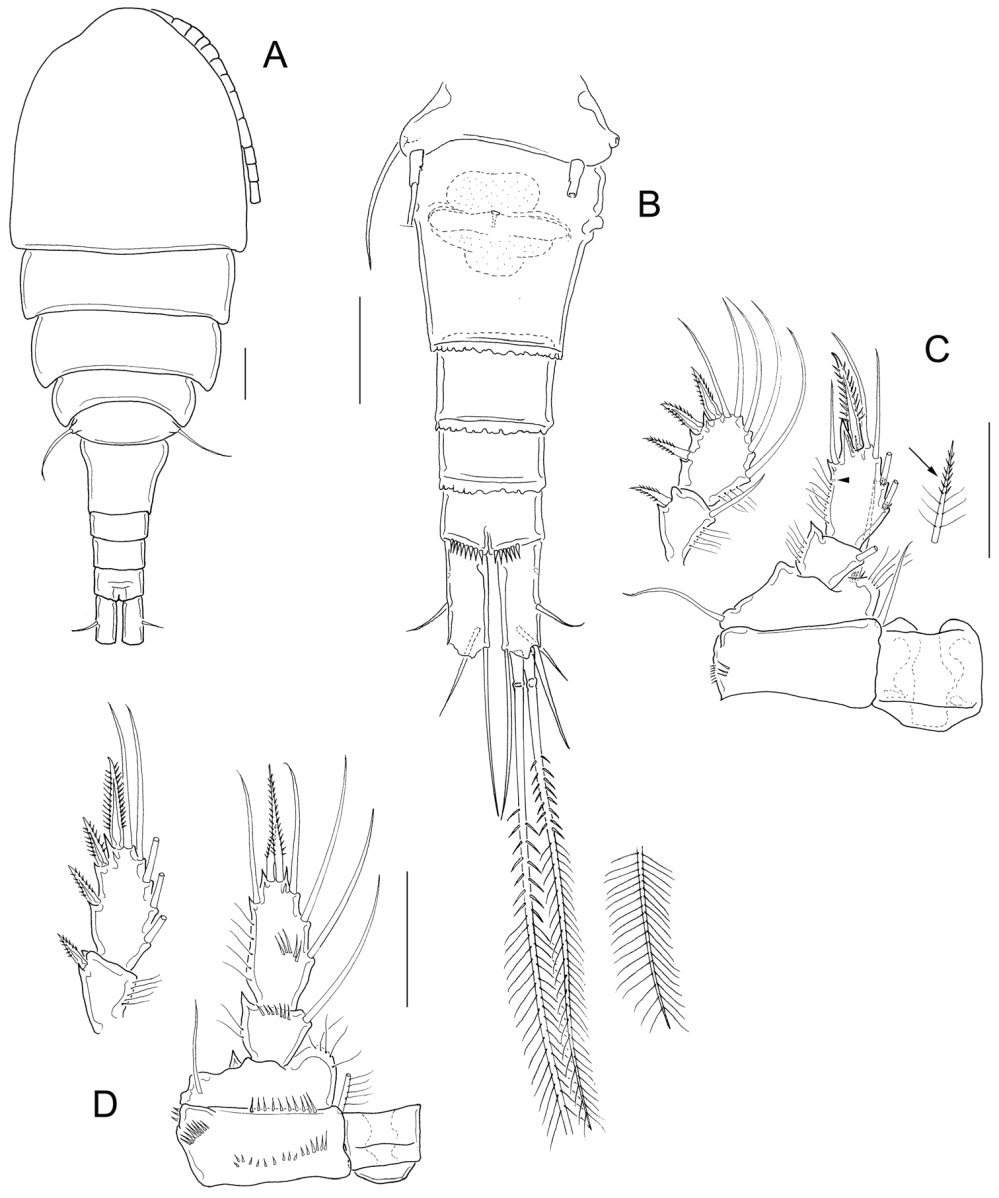
*Microcyclops inarmatus* sp. n. Adult female, holotype (except **A**). **A** Habitus of one paratype specimen (ECOCH-Z-09338) **B** Urosome ventral, note that the last fraction of the inner median terminal caudal seta is separated (ECOCH-Z-09337) **C** P1, caudal (ECOCH-Z-09337) **D** P4, caudal (ECOCH-Z-09337). Scale bars: 50 µm.

**Figure 3.**
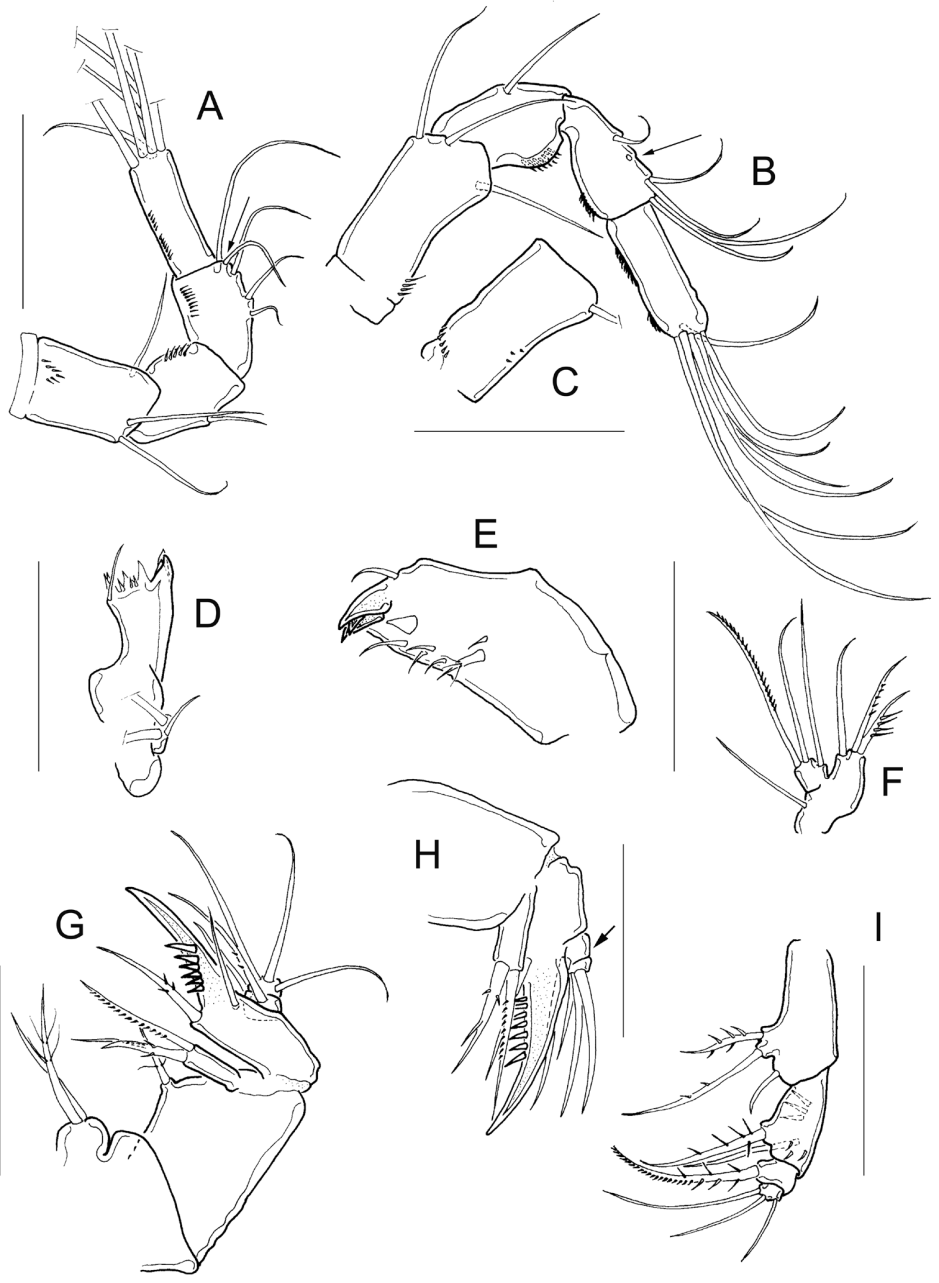
*Microcyclops inarmatus* sp. n. Adult female. **A** Antenna, caudal (USNM-251321), note that the position of missing seta is arrowed **B** Antenna, caudal (ECOCH-Z-09337), note that the position of missing seta is arrowed **C** Antennal basipodite, frontal (ECOCH-Z-09337) **D** Mandible (ECOCH-Z-09337) **E** Maxillule (ECOCH-Z-09337) **F** Maxillular palp (ECOCH-Z-09337) **G** Maxilla (ECOCH-Z-09337) **H** Maxilla (SMNK-2391) **I** Maxilliped (ECOCH-Z-09337). Scale bars: 50 µm.

**Figure 4.**
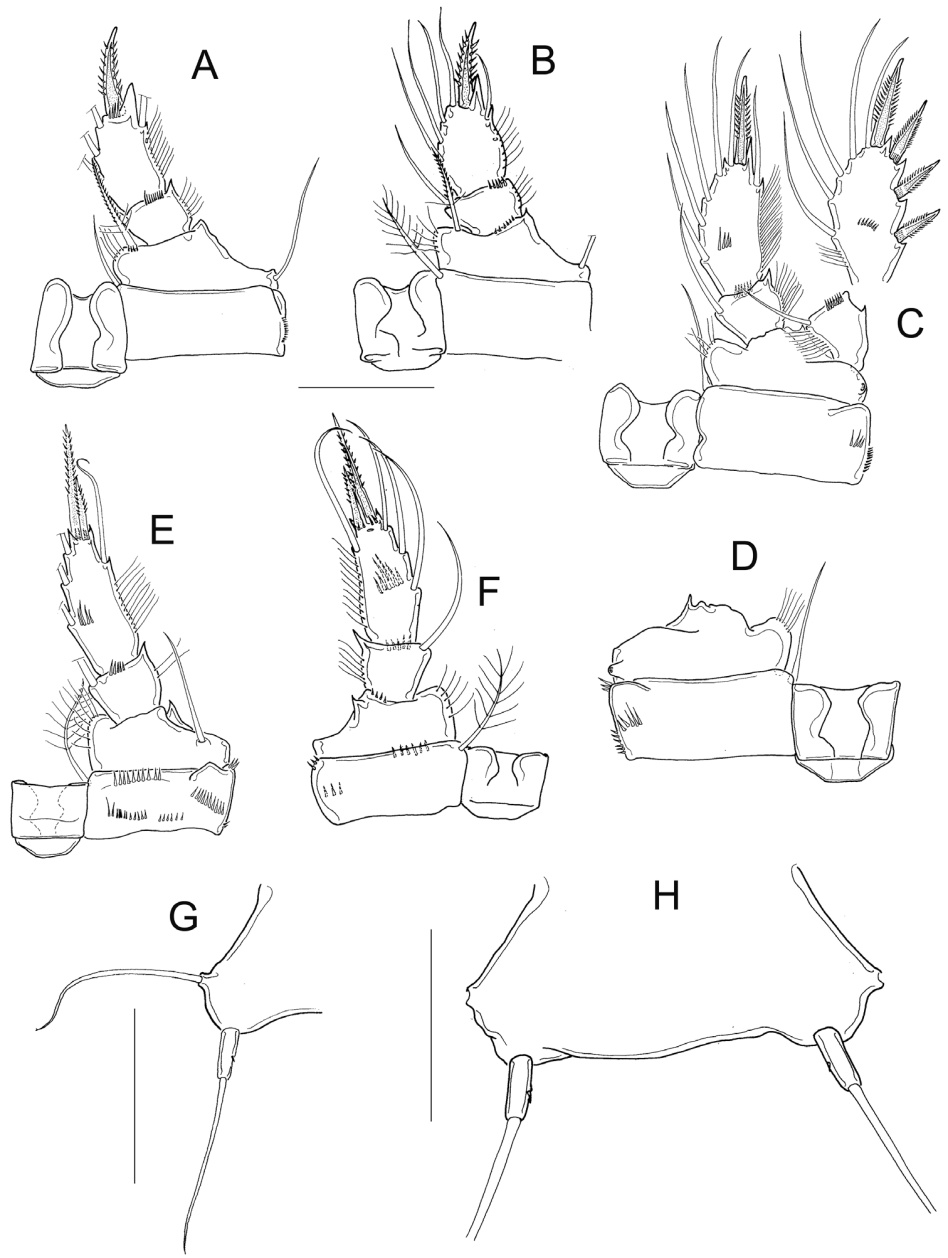
*Microcyclops inarmatus* sp. n. Adult female. **A** P1, frontal (USNM-251321) **B** P1, frontal (SMNK-2391) **C** P2, caudal (ECOCH-Z-09337) **D** P3 coxa, basis, and sclerite, caudal (ECOCH-Z-09337) **E** P4, caudal, Exp unfigured (USNM-251321) **F** P4, frontal, Exp unfigured (SMNK-2391) **G** Fifth pediger and P5 (USNM-251321) **H** Fifth pediger and P5 (SMNK-2392). Scale bars: 50 µm.

**Figure 5.**
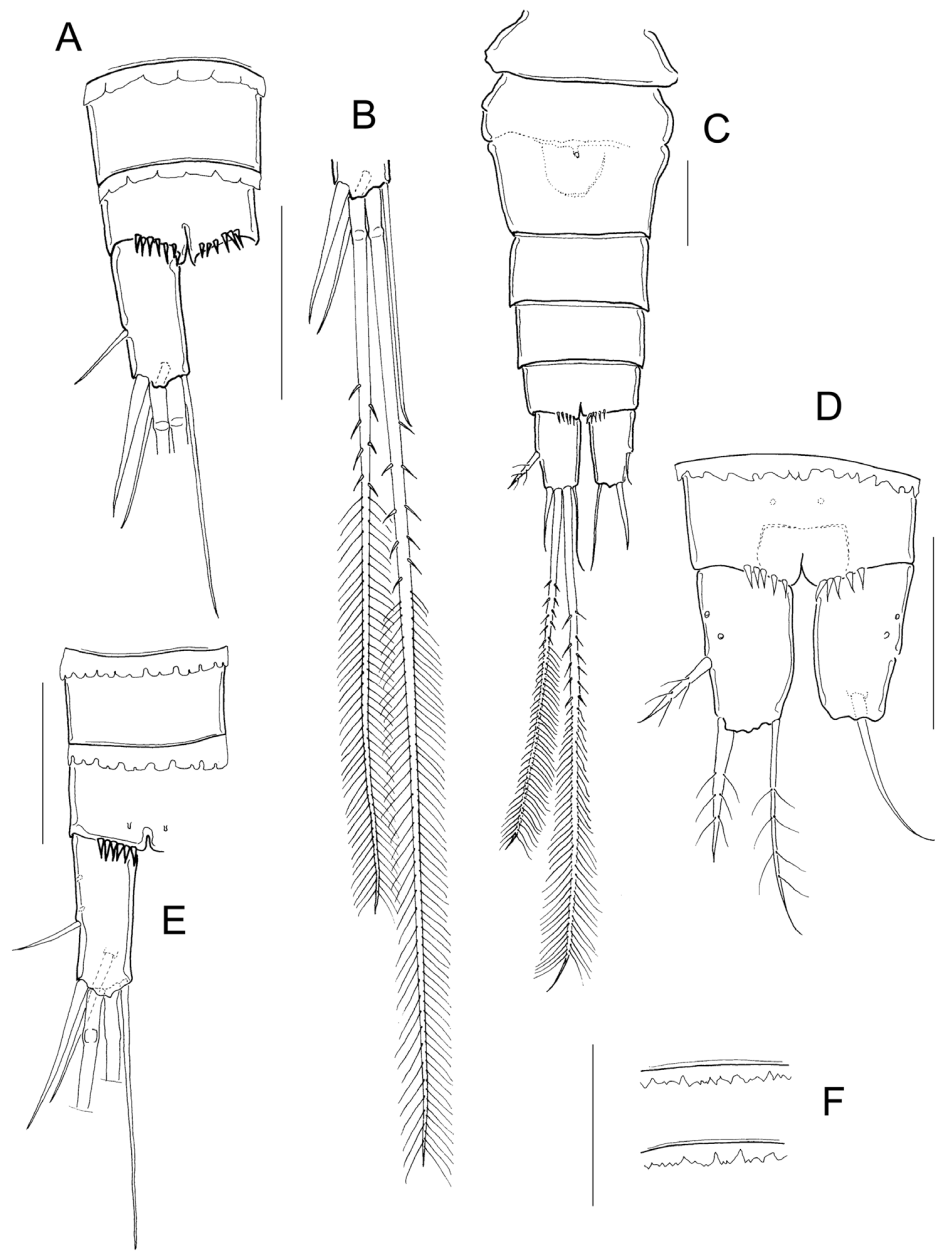
*Microcyclops inarmatus* sp. n. Adult female. **A** Anal somite and caudal rami, ventral (USNM-251321) **B** Terminal caudal setae (USNM-251321) **C** Urosome, ventral (SMNK-2392) **D** Anal somite and caudal rami, ventral (SMNK-2392) **E** Anal somite and caudal rami (ECOCH-Z-0679) **F** Hyaline fringes of urosome (Pajonal). Scale bars: 50 µm.

Figures 2 , 3 , 4 , 5

Microcyclops varicans Reid, 1992; Trans. Am. Microsc. Soc. 111(3), p: 249–250, figs 8d, 9c.

##### Holotype.

One adult female dissected on two slides: A1, A2 (slide 1, ECOCH-Z-09337); mandible, maxillule, maxilla, maxilliped, P1-P4, and urosome (slide 2, ECOCH-Z-09337). Collected 13.I.1998.

##### Paratypes.

10 adult females preserved in 90% ethanol with a drop of glycerine. ECOCH-Z-09338. Collected 13.I.1998.

##### Type locality.

A pond in km 51 lado 1, Villahermosa-Frontera highway 18°23'16"N; 92°47'00"W.

##### Etymology.

the name of the species means un-armed in Latin; it refers to the absence of ornamentation on the intercoxal sclerites, the lack of spinules at base of caudal furcal setae, the reduced number of setae on second antennal endopod, and the reduced ornamentation on antennal basis.

##### Additional material.

One adult female collected 1.02.1935 from Laguna Rincon, Haiti (slide SMNK-2391; labelled as *Microcyclops dubitabilis* with A1, maxilla, P1-P4). One adult female collected from Laguna Rincon, Haiti (slide SMNK-2392; labelled as *Microcyclops dubitabilis* with urosome).

One adult female collected 05.1986 from Shark river slough, Everglades National Park, Florida, USA (slide 2 of 7, USNM-251321; labelled as *Microcyclops varicans* with A1, A2, P1-P4, and urosome).

##### Diagnosis.

Adult female: Dorsal margin of prosomal somites smooth; body length 565 to 615 µm in paratypes. Antennule 12-segmented, not reaching the distal margin of the first prosomal segment (Fig. 2A). Fifth pediger nude; cylindrical free segment of P5 more than 3 times as long as wide, with tiny inner spine; genital double somite expanded proximally. Anal somite with strong spines on ventral distal margin; length to width ratio of caudal ramus less than 3; no spinules at base of lateral and outermost terminal caudal setae (Fig. 2B). Outer median terminal and inner median terminal caudal setae with heteronomous setulation (Fig. 2B). Endopodites and exopodites of P1-P4 bisegmented with setation formula as in Table 1, inner basis of P1 with long spine (long arrow in Fig. 2C), Enp2P1 with one pore on lateral margin (short arrow in Fig. 2C). Intercoxal sclererites of P1-P4 unarmed, long setules on inner margin of basipodites of P1-P4, medial spine of Enp2P4 almost as long as the segment and twice the length of the lateral spine (Fig. 2D).

Adult male: unknown.

##### Description of female.

*Antennule* 12-segmented; antenna with 3-segmented Enp armed with 1, 6, and 7 setae respectively (Fig. 3A, B–position of missing setae in specimens is arrowed). Antennal basis with one group of spinules on the basal-outer margin in caudal view (Fig. 3A, B); frontal surface of the antennal basis with two rows of tiny spinules (Fig. 3C). Nine teeth on mandibular gnathobase (Fig. 3D). Maxillule (Fig. 3E) with unarmed palp; apical region of maxillular palp with two armed setae plus one smooth seta, three setae (one armed) on lateral lobe, proximal seta smooth (Fig. 3F). Distal coxal endite of the maxilla with two long setae: the proximal seta with two tiny spines at its base and bifurcated, distal seta with one row of tiny spines along one margin (Fig. 3G, H). Basipodite with one claw-like projection bearing 5-7 strong spines on the concave margin and one long, armed seta on its base; two-segmented Enp bearing 2 and 3 setae respectively (Fig. 3G). Because of the condition of the microscope slide preparatum we could not verify one basal seta on maxillar Enp1 (arrowed in Fig. 3H). Maxilliped with syncoxa (3 setae), basis (2 setae), and two-segmented Enp bearing 1 and 3 setae. Basis of maxilliped with a row of spinules on frontal and caudal surfaces (Fig. 3I).

*Basipodites* of P1−P3 with long hair-like setules on the inner margins; one row of tiny spinules along the lateral margins of coxa; intercoxal sclerites naked (Fig. 4A–D). Basis of P1 with one long spine on inner margin; spine reaching distal middle of Enp2P1 and armed with heteronomous setulation: hair-like setules on its base, tiny spinules distally (Figs 2C; 4A, B). One pore on the lateral margin of Enp2P1 (Fig. 4A, B). Basipodite of P4 with long hair-like setules on inner margin; P4 intercoxal sclerite quadrangular (Figs 2D; 4E, F), Enp2P4 2.14 ± 0.2 times as long as wide; and medial spine 1.97 ± 0.25 as long as lateral spine and 0.91 ± 0.04 as long as the segment (Figs 2D; 4E, F).

*Fifth pediger* nude; P5 with one cylindrical free segment, 3.23 ± 0.4 times as long as wide, bearing one tiny medial spinule. Free segment 0.27 ± 0.01 times as long as distal seta (Figs 2B; 4G, H). Hyaline fringes of urosomites serrated (Fig. 2B), petaloid or rounded (Fig. 5 A, C–F). Length to width ratio of caudal ramus 2.54 ± 0.44, inner margin naked; no spinules at base of lateral caudal (II) and outermost terminal caudal setae (III) (Fig. 2B). Only 5-8 strong spinules present ventrally on the distal margin of anal somite, no spinules dorsally (Fig. 5 A, C–E). Lateral caudal seta (II) inserted at 58.6 ± 3.9% of caudal ramus.

Dorsal caudal seta (VII) 0.9 ± 0.1 times as long as caudal ramus, and innermost terminal caudal seta (VI) 1.4 ± 0.04 times as long as caudal rami (Fig. 2B). Relative lengths of terminal caudal seta from outermost caudal seta to innermost caudal seta is 1.0 : 4.9 : 7.3 : 1.6 (Figs 2B; 5B, C). Outer median terminal caudal seta (IV) and inner median terminal caudal seta (V) with heteronomous setulation: proximally with spinule-like setules and distally with long and fine setules (Figs 2B; 5B, C).

#### Microcyclops dubitabilis

Taxon classificationAnimaliaCyclopoidaCyclopidae

Kiefer, 1934

Figures 6 , 7 , 8

##### Description of female.

**Figure 6.**
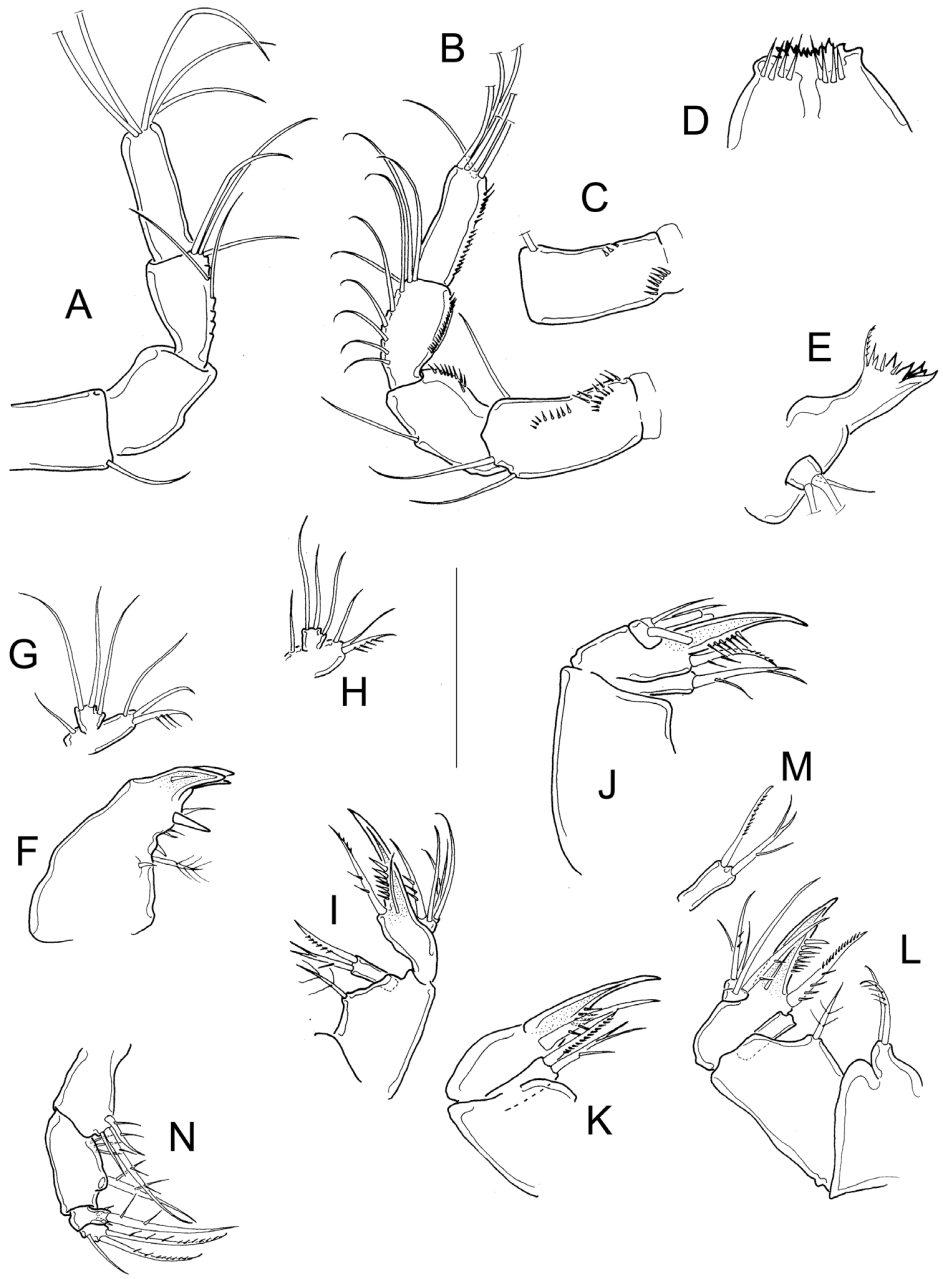
*Microcyclops dubitabilis* Kiefer, 1934. Adult female. **A** Antenna, frontal (SMNK-2204) **B** Antenna, caudal (km 51-1) **C** Antennal basipodite, frontal (km 51-1) **D** Labrum (km 51-1) **E** Mandible (km 51-1) **F** Maxillule (km 51-1) **G** Maxillular palp (km 51-1) **H** Maxillular palp (USNM-251322) **I** Maxilla (USNM-251322) **J** Maxilla (SMNK-2081) **K** Maxilla (SMNK-2204) **L** Maxilla (km 51-1) **M** Distal coxal endite **N** Maxilliped (km 51-1). Scale bar: 50 µm.

*Antennule* 11, or 12-segmented (intra- and interpopulation variation); 3-segmented endopod of antenna bearing 1, 9, and 7 setae, respectively (Fig. 6A, B). Antennal basis with three long rows of spinules on caudal surface (Fig. 6B): two basal rows, and one median row; frontal surface of antennal basis with two rows of tiny spinules (Fig. 6C). Labrum with 6−7 teeth between two curved lateral teeth, and 3−4 strong spinules on each round projections of the plate (Fig. 6D). Eight teeth on mandibular gnathobase (Fig. 6E). *Maxillule* as in Fig. 6F, maxillular palp with one armed seta plus two smooth setae apically, three smooth setae on lateral lobe, and one proximal nude seta (Fig. 6G, H). Maxilla with armed setae on distal coxal endite: proximal seta with one long spine-like setule at its base and bifurcated apically, distal seta with one row of tiny spines along inner margin (Fig. 6I–K, M). *Basipodite* with claw-like projection bearing 6−8 thin spinules on concave margin and one long seta on its base; this seta armed with two rows of spinules (long spinules on inner margin, and short spinules on outer margin) (Fig. 6I–L) . Maxilla with two-segmented Enp bearing 2 and 3 setae respectively (Fig. 6I–L). Maxilliped with syncoxa (3 setae), basis (2 setae), and two-segmented Enp bearing 1 and 3 setae, respectively. Basis of the maxilliped nude, two spinules present on frontal surface of Enp1 (Fig. 6N).

**Figure 7.**
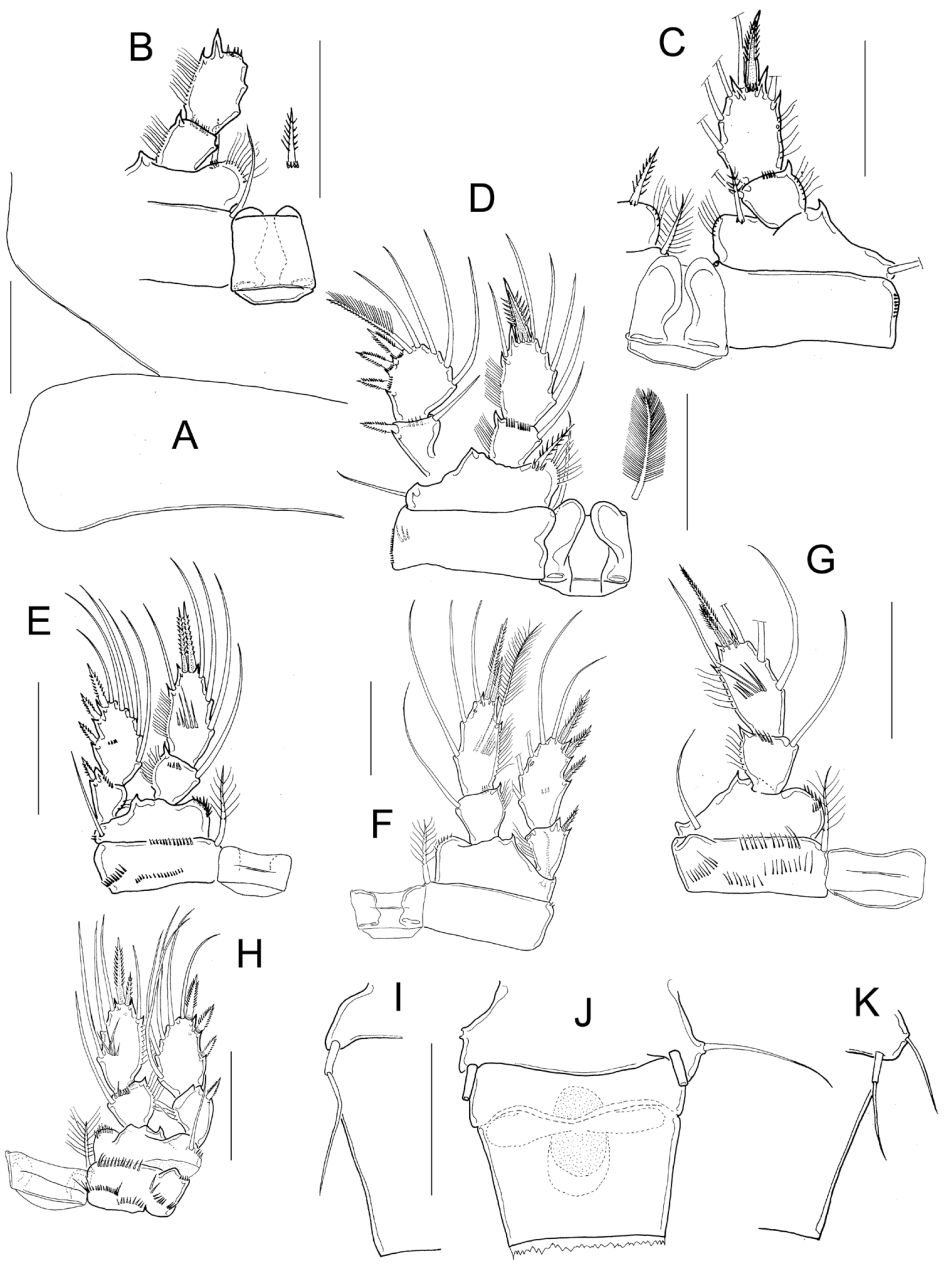
*Microcyclops dubitabilis* Kiefer, 1934. Adult female. **A** First and second prosomal somite, dorsal (SMNK-2189) **B** P1, caudal (USNM-251322) **C** P1, frontal (SMNK-2081) **D** P1, frontal (km 51-1) **E** P4, caudal (USNM-251322) **F** P4, frontal (SMNK-2189) **G** P4, caudal (MNHN-Cp6764) **H** P4, caudal (km 51-1) **I** Fifth pediger, P5, genital double somite, ventral (USNM-251322) **J** Fifth pediger, P5, genital double somite, ventral (MNHN-Cp5398) **K** Fifth pediger, P5, genital double somite, ventral (SMNK-2204). Scale bars: 50 µm.

Dorsal margin of *prosomal somites* smooth (Fig. 7A). Basis of P1 medially hairy. One short spine present on inner margin, spine biserially armed with spinule-like setules (homonomous ornamentation) and reaching slightly beyond distal margin of the Enp1P1. Intercoxal sclerite of P1 naked (Fig. 7B–D). Pore on lateral margin of Enp2P1 sometimes present (interpopulation variation). Inner margin of basis of P2 and P3 hairy, and intercoxal sclerites of these swimming legs naked (unfigured). Inner margin of P4 basis with short hairs; intercoxal sclerite naked, rectangular (Fig. 7E–H), Enp2P4 1.9 ± 0.1 times as long as wide; medial spine 1.8 ± 0.3 times as long as lateral spine, and 0.8 ± 0.1 times as long as segment. Apical spines of Enp2P4 are subequal only in female USNM-251322 (Fig. 7E).

**Figure 8.**
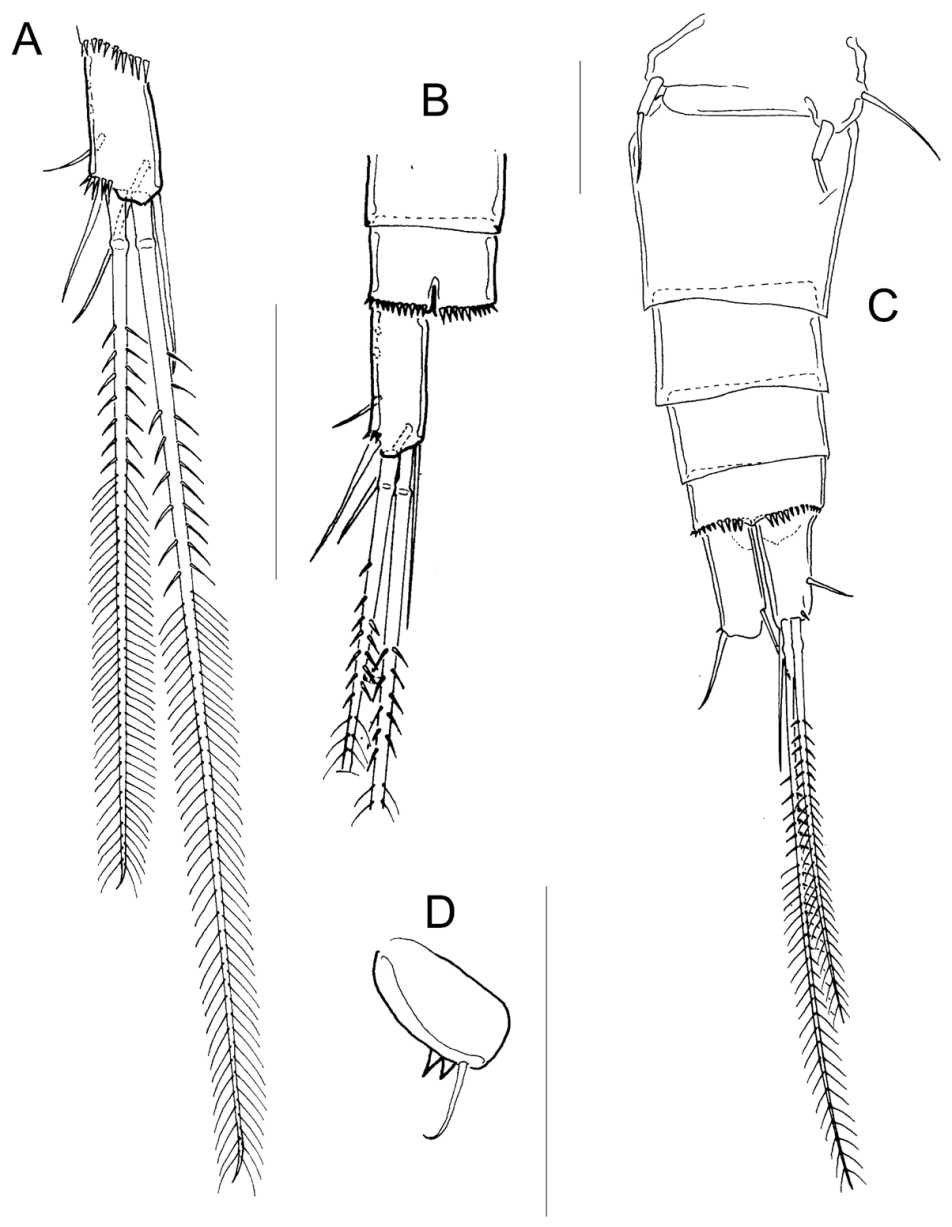
*Microcyclops dubitabilis* Kiefer, 1934. Adult female. **A** Caudal rami, ventral (USNM-251322) **B** Anal somite and caudal rami, ventral (SMNK-2204) **C** Urosome, ventral (SMNK-2081) **D** P6 (km 51-1). Scale bars: 50 µm.

*Fifth pediger* nude; P5 free segment cylindrical, 3.6 ± 0.8 times as long as wide, without inner spine. Free segment 0.4 ± 0.1 times the length of the distal seta (Figs 7I–K; 8C). Length to width ratio of caudal ramus 2.4 ± 0.2, inner margin naked. With or without spines at base of seta II (intrapopulation variation), spinules always present at base of setae III. Distal margin of anal somite bearing spinules: medial spinules are longer than lateral ones on ventral surface; spinule row can extend laterally or dorsally (Fig. 8 A–C). Seta II inserted at 71 ± 5.7% of caudal ramus.

*Seta* VII 1.02 ± 0.3 times as long as caudal ramus, and seta VI 1.4 ± 0.2 times longer than caudal ramus. Relative lengths of terminal caudal seta from outermost caudal seta to innermost caudal seta are 1.0 : 4.9 : 7.1 : 1.6 (Fig. 8A, C). Seta IV and seta V with heteronomous setulation: proximally with spine-like setules and distally with long and fine setules (Fig. 8A–C). Sixth leg with two medial spines and one lateral seta (Fig. 8D).

#### Microcyclops ceibaensis

Taxon classificationAnimaliaCyclopoidaCyclopidae

(Marsh, 1919)

Figures 9 , 10 , 11

##### Description of female.

**Figure 9.**
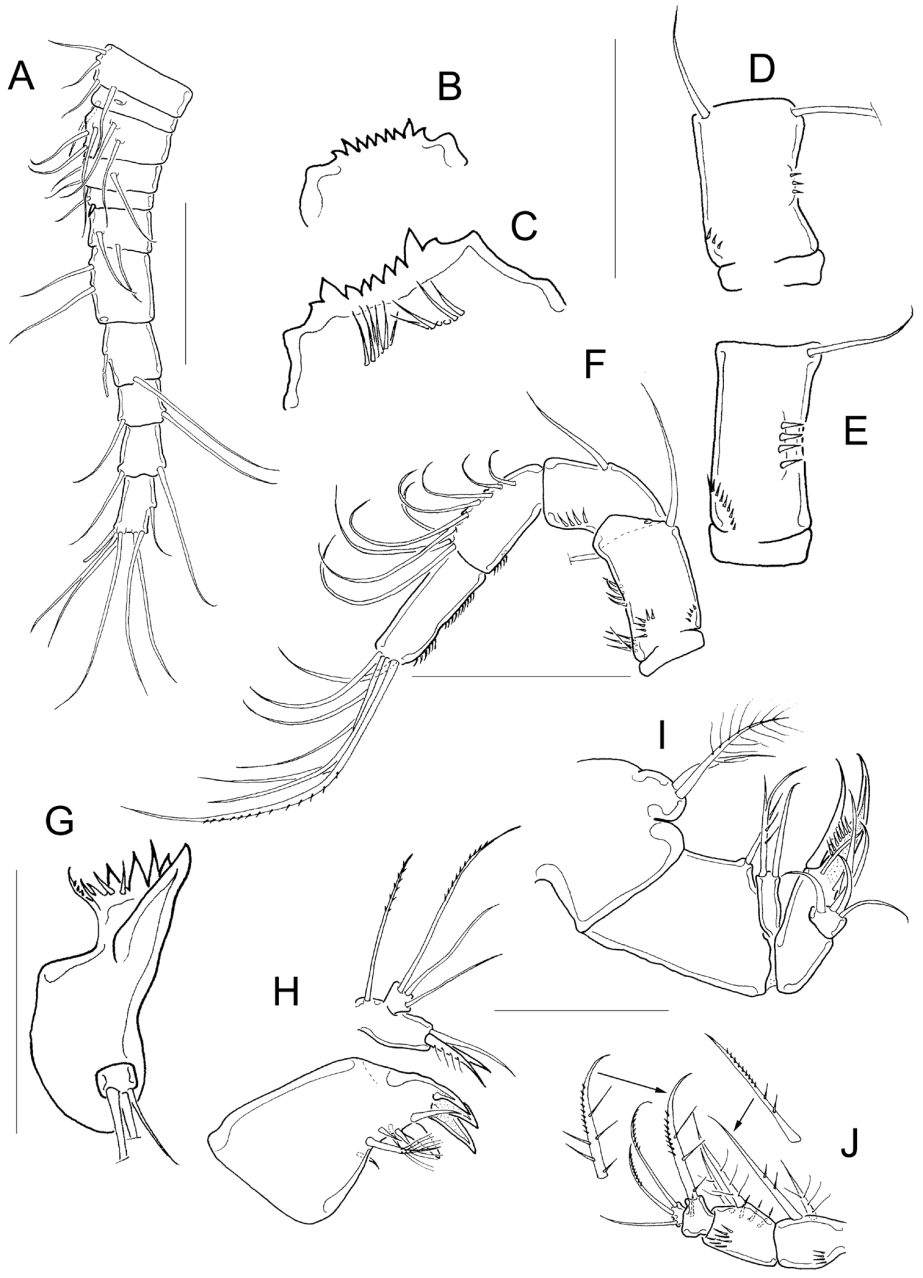
*Microcyclops ceibaensis* (Marsh, 1919). Adult female. **A** Antennule, segments 2-12 (USNM-222299) **B** Labrum (USNM-222299) **C** Labrum (km 51-2) **D** Antennal basipodite, frontal (USNM-222299) **E** Antennal basipodite frontal (km 154) **F** Antenna, caudal (km 154) **G** Mandible (km 51-1) **H** Maxillule (km 154) **I** Maxilla (km 51-2) **J** Maxilliped (km 154). Scale bars: 50 µm.

*Antennule* 12-segmented (Fig. 9A). Antenna with 3-segmented endopod with 1, 9, and 7 setae, respectively (Fig. 9F). Frontal surface of antennal basis with one basal row of spinules arranged in arc next to medial (inner) margin, and one median row of spinules next to lateral (outer) margin. (Fig. 9D, E). Caudal surface of antennal basis with two basal rows of spinules arranged in arc, plus two rows of long spinules on outer margin (Fig. 9F). Labrum with 7 marginal teeth between two lateral curved teeth, and two rows of long spinules (6) overhanging distal margin (Fig. 9B, C). Gnathobase of the mandible with eight teeth (Fig. 9G). Maxillular palp with three apical setae, three setae on lateral lobe, and one proximal seta. The proximal seta armed on both margins, one seta on lateral lobe and one apical seta with setules (Fig. 9H). Distal coxal endite of the maxilla with two long setae: the proximal seta with two long basal spinules and bifurcated apically, distal seta smooth (Fig. 9I). Basipodite with one claw-like projection bearing thin spinules on concave margin, and one long smooth seta on its base. One-segmented Enp bearing 5 setae (Fig. 9I). Maxilliped with syncoxa (2 setae), basis (2 setae), and two-segmented Enp bearing 1 and 3 setae, respectively. Ornamentation of setae on syncoxa and Enp1 variable (intrapopulation variation, arrowed in Fig. 9J). Syncoxa and basis of maxilliped with a row of spinules on caudal surface next to lateral margin (Fig. 9J).

**Figure 10.**
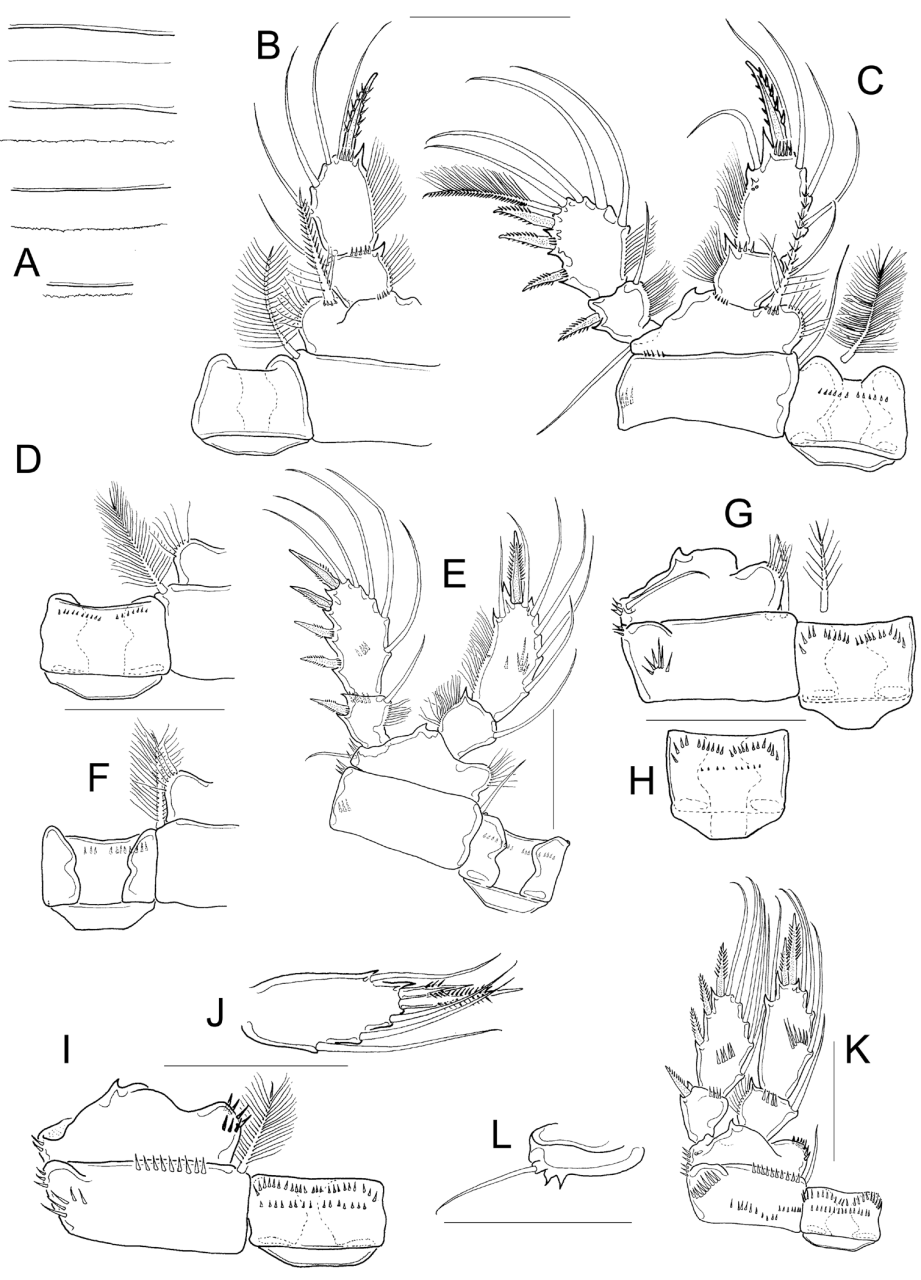
*Microcyclops ceibaensis* (Marsh, 1919). Adult female. **A** Prosomal fringes, dorsal **B** P1, frontal (USNM-222299) **C** P1, caudal (km 51-2) **D** P2 intercoxal sclerite, inner coxa and basis, caudal (USNM-222299) **E** P2, frontal (km 51-2) **F** P3 sclerite, inner coxa and basis, frontal (USNM-222298) **G** P3 intercoxal sclerite, coxa and basis, caudal (km 51-1) **H** P3 intercoxal sclerite, caudal (km 51-2) **I** P4 intercoxal sclerite, coxa and basis, caudal (USNM-222299) **J** Enp3P4 (USNM-222299) **K** P4, caudal (km 51-2) **L** P6. Scale bars: 50 µm.

Dorsal margin of *prosomal somites* slightly serrated (Fig. 10A). Basis of P1−P3 with pilose inner margin. Intercoxal sclerites of P1-P3 with one row of short spinules (Fig. 10B-G), in some populations the sclerite of P3 with two rows of spinules (Fig. 10H). Enp2P1 with two pores on lateral margin (Fig. 10C). Because of the condition of the specimen in slide USNM-222299, it was not possible to verify the presence of spinules on the sclerite as well as the pores on the second endopodal segment of P1 (Fig. 10B).

*P1 basis* with long medial spine reaching distal third of Enp2P1. Spine ornamented with long setules near base and with short spinule-like setules more distally (Fig. 10B, C). Inner margin of P4 basis with strong spinules. Intercoxal sclerite rectangular, and ornamented with two rows of spinules (Fig. 10I, K). Enp2P4 2.2 ± 0.1 times as long as wide; medial spine 1.5 ± 0.1 times as long as the lateral spine, and 0.6 ± 0.06 times as long as segment (Fig. 10J, K). Sixth leg with one long seta plus two short spines (Fig. 10L).

**Figure 11.**
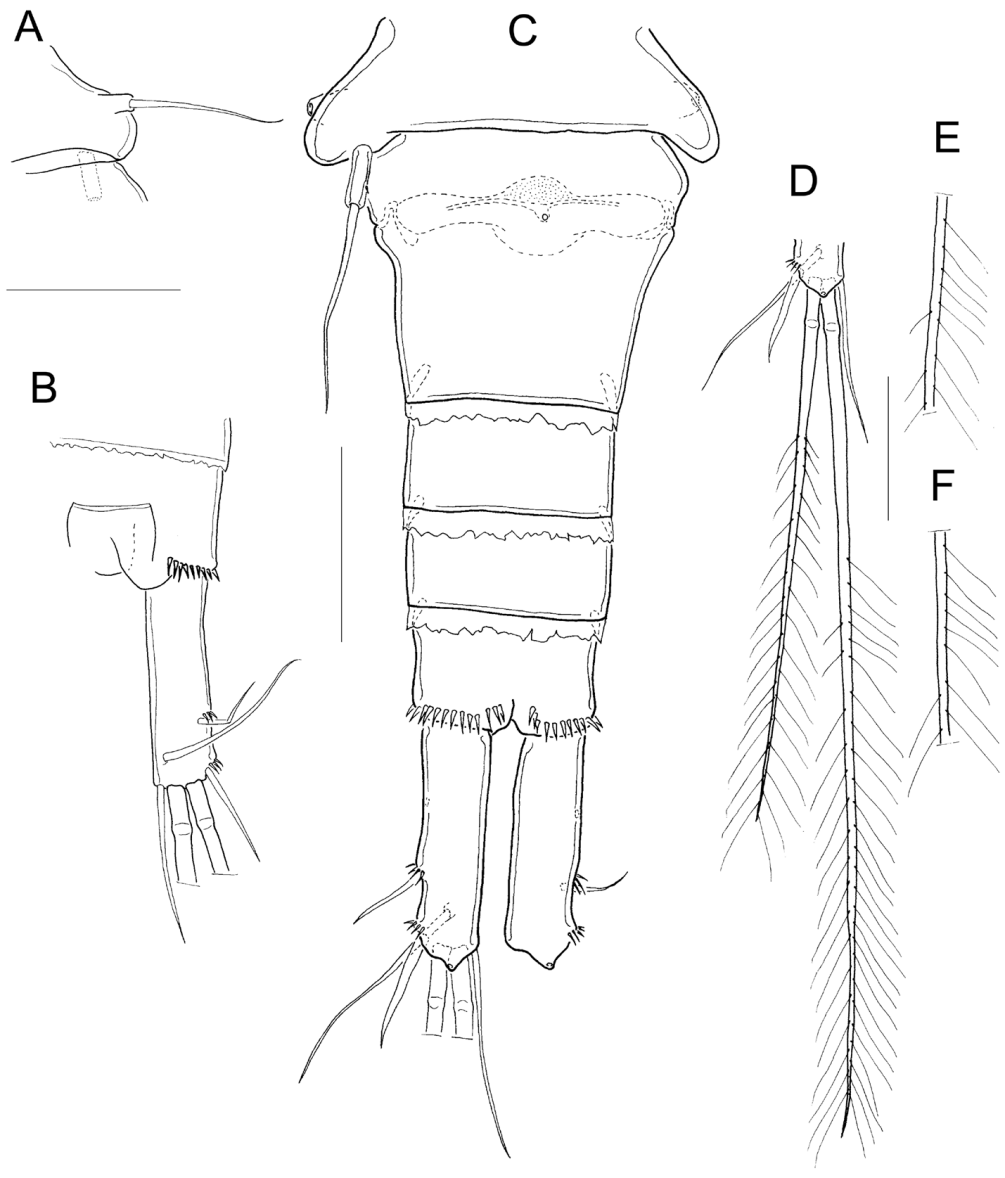
*Microcyclops ceibaensis* (Marsh, 1919). Adult female. **A** Fifth pediger, dorsal (USNM-222299) **B** Anal somite and caudal rami, dorsal (USNM-222299) **C** Urosome, ventral (km 51-1) **D** Caudal ramus and caudal setae, ventral (km 51-1) **E** Detail of inner median caudal seta (km 154) **F** Detail of inner median caudal seta (km 51-1). Scale bars: 50 µm.

*Fifth pediger* nude (Fig. 11A). Urosomal somites with serrated hyaline fringes (Fig. 11B, C). Fifth leg with one cylindrical free segment 3.8 ± 1.4 times as long as wide; tiny spinule present on inner margin. P5 free segment 0.3 ± 0.1 times the length of the distal seta (Fig. 11C). Distal margin of anal somite with a continuous row of strong spinules on ventral and dorsal surfaces (Fig. 11B, C). Caudal ramus 3.6 ± 0.4 times longer than wide, inner margin naked. Spinules present at base of caudal setae II and III; seta II inserted at 69 ± 3.2% of the caudal ramus (Fig. 11C).

*Seta VII* 0.7 ± 0.1 times as long as caudal ramus, seta VI 0.8 ± 0.1 times as long as caudal ramus. Relative lengths of terminal caudal seta from outermost caudal seta to innermost caudal seta: 1.0 : 5.7 : 9.5 : 1.8 (Fig. 11C, D). Seta IV and seta V with homonomous setulation, with long and fine setules at whole length (Fig. 11D); inner median terminal caudal seta (V) with interrupted row of setules along the proximal, lateral margin (Fig. 11E, F).

#### Microcyclops echinatus

Taxon classificationAnimaliaCyclopoidaCyclopidae

Fiers, Ghenne & Suárez-Morales, 2000

Figures 12 , 13

##### Description of female.

**Figure 12.**
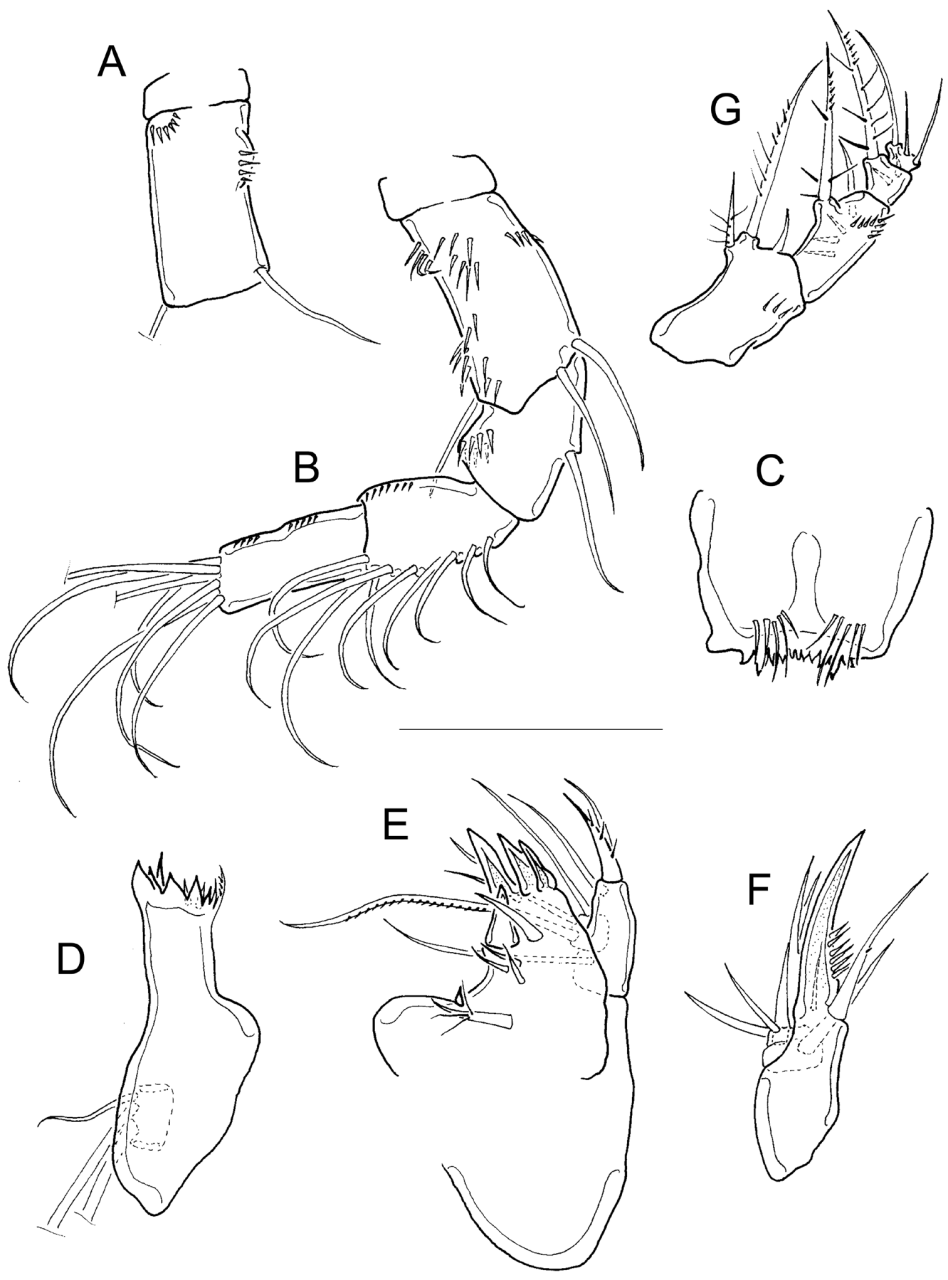
*Microcyclops echinatus* (Fiers et al., 2000). Adult female (km 51-2). **A** Antennal basipodite, frontal **B** Antenna, caudal **C** Labrum **D** Mandible **E** Maxillule **F** Maxilla **G** Maxilliped. Scale bar 50 µm.

This description is a complement to the original description of Fiers et al. (2000). Frontal surface of antennal basis with one basal, inner row of spinules arranged in an arc, and one longitudinal row of spinules near lateral margin (Fig. 12A). Antenna with three-segmented endopod bearing 1, 9, and 7 setae, respectively (Fig. 12B). Caudal surface of antennal basis with two rows of long spinules next to exopodal seta, one group of long spinules at basal position, one basal row on inner margin and another basal row on outer margin (Fig. 12B). Nine teeth on the distal margin of the labrum (Fig. 12C). Eight teeth present on gnathobase of mandible (Fig. 12D). Maxillular palp with three apical setae (one of these setae armed with long setules); lateral lobe with three setae, the longer seta armed; proximal seta nude (Fig. 12E). Maxillar basipodite with one claw-like projection bearing thin spines on concave margin and one long seta with one (Fig. 12F) or four tiny spinules (Fiers et al. 2000); maxilla with two-segmented Enp bearing 2 and 3 setae on first and second endopodal segments, respectively (Fig. 12F). Maxilliped with syncoxa (3 setae), basis (2 setae), and two-segmented Enp bearing 1 and 3 setae. Syncoxa and basis of maxilliped with rows of spinules on caudal surface (Fig. 12G).

**Figure 13.**
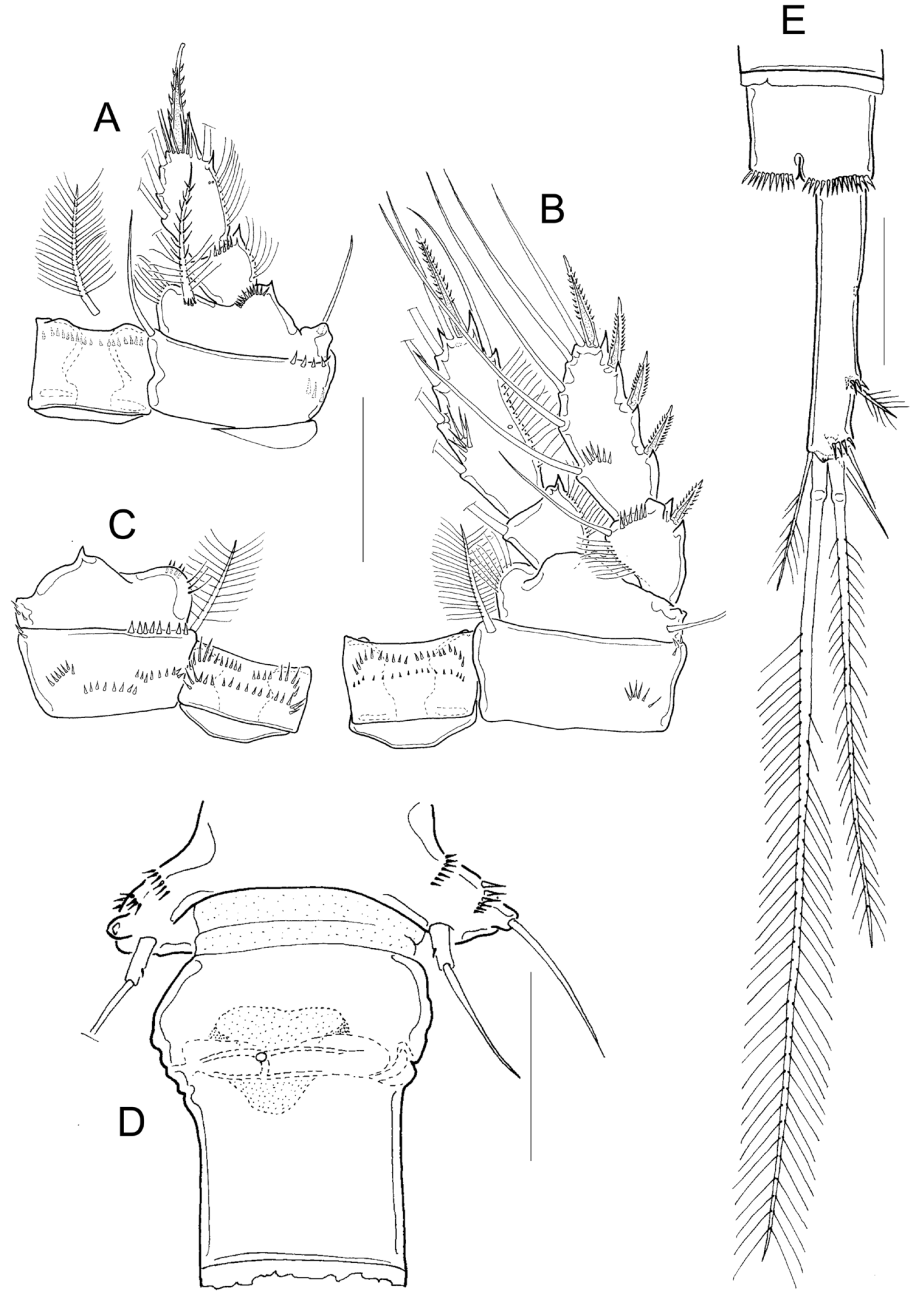
*Microcyclops echinatus* (Fiers et al., 2000). Adult female (Guanal). **A** P1, frontal **B** P3, caudal **C** P4 coxa, basis, sclerite, caudal **D** Fifth pediger, P5 and genital double-somite, ventral **E** Anal somite, caudal rami, and caudal setae, ventral. Scale bars: 50 µm.

Two pores on lateral margin of second endopodal segment of P1, very long spinules present at insertion of apical spine of Enp2P1. Long medial spine of P1 basis with heteronomous setulation (Fig. 13A). Inner margin of P1−P3 basis with long hair-like setae (Fig. 13A, B), inner margin of P4 basis with one row of tiny spinules and one row of long setules (Fig. 13C). Intercoxal sclerites of all swimming legs ornamented on caudal surface: P1 with one row of spinules and P2 to P4 with two rows of spinules (Fig. 13A–C). Enp2P4 2.5 ± 0.1 times as long as wide; medial spine is 2.0 ± 0.1 times as long as lateral spine, and 0.8 ± 0.1 times as long as the segment.

*Fifth pediger* with rows of spinules on ventro-lateral surfaces. Fifth leg with tiny spinule on inner margin (Fig. 13D); cylindrical free segment 3.7 ± 0.1 times longer than wide and 0.45 ± 0.01 times as long as distal seta of P5. Caudal ramus 5.9 ± 0.4 times longer than wide. Seta VII 0.5 ± 0.1 times as long as caudal ramus, seta VI 0.5 ± 0.05 times as long as caudal ramus. Relative lengths of terminal caudal seta from outermost to innermost caudal seta, 1.0 : 6.5 : 10.4 : 2.0. Seta IV and seta V with homonomous setulation, bearing long and fine setules (Fig. 13E).

#### Microcyclops finitimus

Taxon classificationAnimaliaCyclopoidaCyclopidae

Dussart, 1984

Figure 14

##### Description of female.

**Figure 14.**
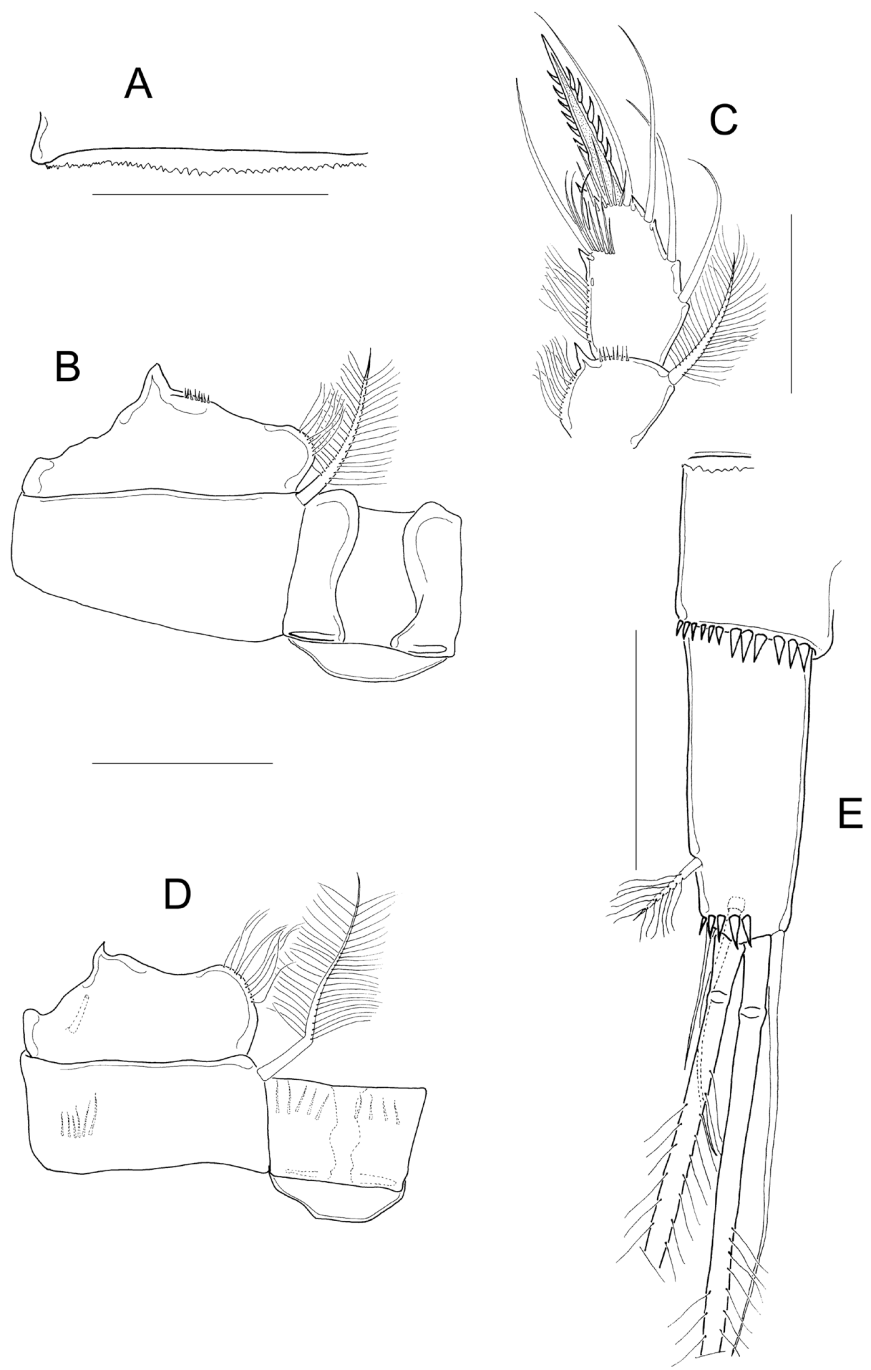
*Microcyclops finitimus* Dussart, 1984. Adult female (MNHN-Cp7294). **A** Fifth pediger and hyaline fringe, dorsal **B** P1, coxa, basis and sclerite, frontal **C** P1, Enp, frontal **D** P4, coxa, basis and intercoxal sclerite, frontal **E** Anal somite, caudal rami, and caudal setae, ventral. Scale bars: 50 µm.

The following description is complementary to the original description of Dussart (1984). Antennule 12-segmented. Dorsal margin of prosomal somites 1 to 3 smooth (unfigured); hyaline fringe of fifth pediger serrated dorsally (Fig. 14A). Intercoxal sclerite of P1 smooth, inner margin of P1 basis with long hair-like setules, without spine on inner margin (Fig. 14B). Enp2P1 with one pore on lateral margin. Row of long spinules present at base of apical spine and lateral seta of Enp2P1 (Fig. 14C). Inner margin of P4 basis with long setules, intercoxal sclerite quadrangular, with one row of long spinules on caudal surface (Fig. 14D). Enp2P4 2.2 times as long as wide; medial spine 1.4 times as long as lateral spine, and 0.8 times as long as segment.

*Anal somite* with continuous row of spinules along distal margin (ventrally and dorsally), but on ventral surface medial spinules are longer and stronger than lateral spinules (Fig. 14E). No spinules at base of caudal seta II, but spinules present at base of caudal seta III; length to width ratio of caudal ramus 2.7. Relative lengths of terminal caudal setae from outermost to innermost seta, 1.0 : 6.1 : 8.9 : 2.1. Caudal setae IV, and V with homonomous setulation, bearing long and fine setules (Fig. 14E). Dorsal caudal seta (VII) 0.7 times as long as caudal rami, and innermost caudal seta (VI) 1.2 times longer than caudal rami. Lateral caudal seta (II) inserted at 75.5% of caudal ramus length.

#### Microcyclops anceps anceps

Taxon classificationAnimaliaCyclopoidaCyclopidae

(Richard, 1897)

**Figure 15.**
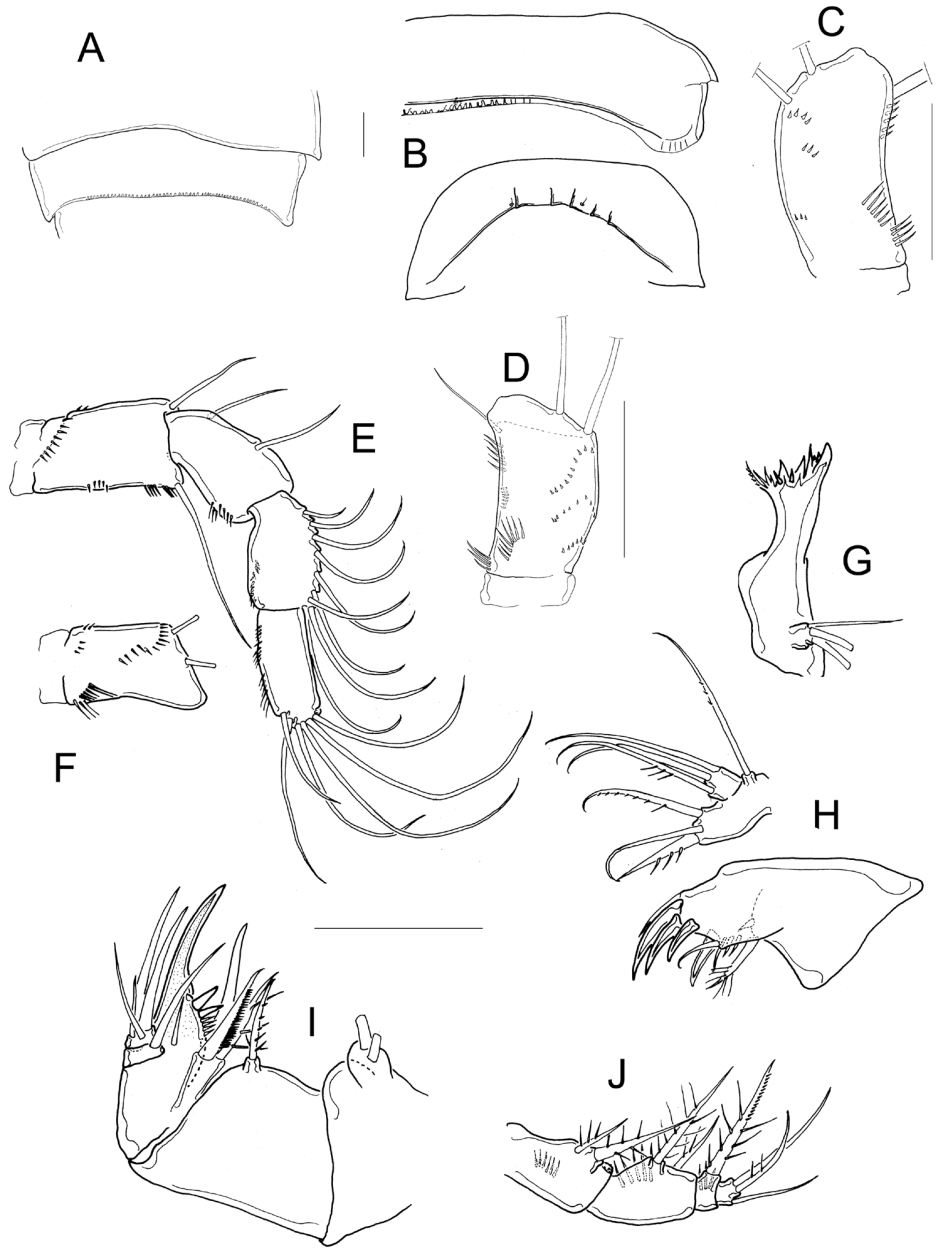
*Microcyclops anceps anceps* (Richard, 1897). Adult female. **A** Second prosomal somite, dorsal (SMNK-2832) **B** Second and fourth prosomal somites, dorsal (Matillas) **C** Antennal basipodite, caudal (MNHN-Cp6876) **D** Antennal basipodite, caudal (MNHN-Cp7296) **E** Antenna, frontal (Matillas) **F** Antennal basipodite, caudal (Matillas) **G** Mandible (Matillas) **H** Maxillule (Matillas) **I** Maxilla (Matillas) **J** Maxilliped (Matillas). Scale bars: 50 µm.

**Figure 16.**
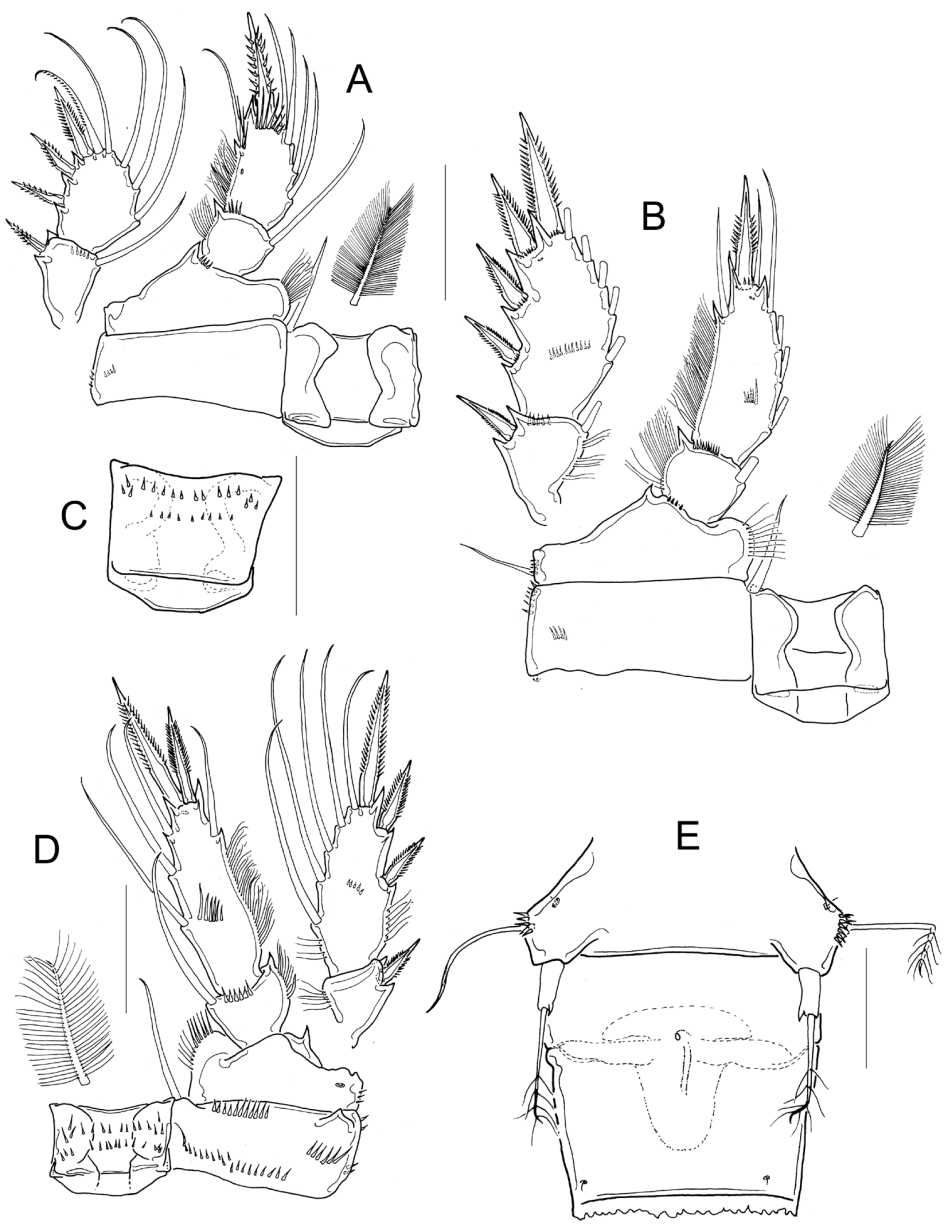
*Microcyclops anceps anceps* (Richard, 1897). Adult female. **A** P1, frontal (Pajonal) **B** P2, frontal (Pajonal) **C** P3 intercoxal sclerite, caudal (Pajonal) **D** P4, caudal (Pajonal) **E** Fifth pediger, genital double-somite (Matillas). Scale bars: 50 µm.

**Figure 17.**
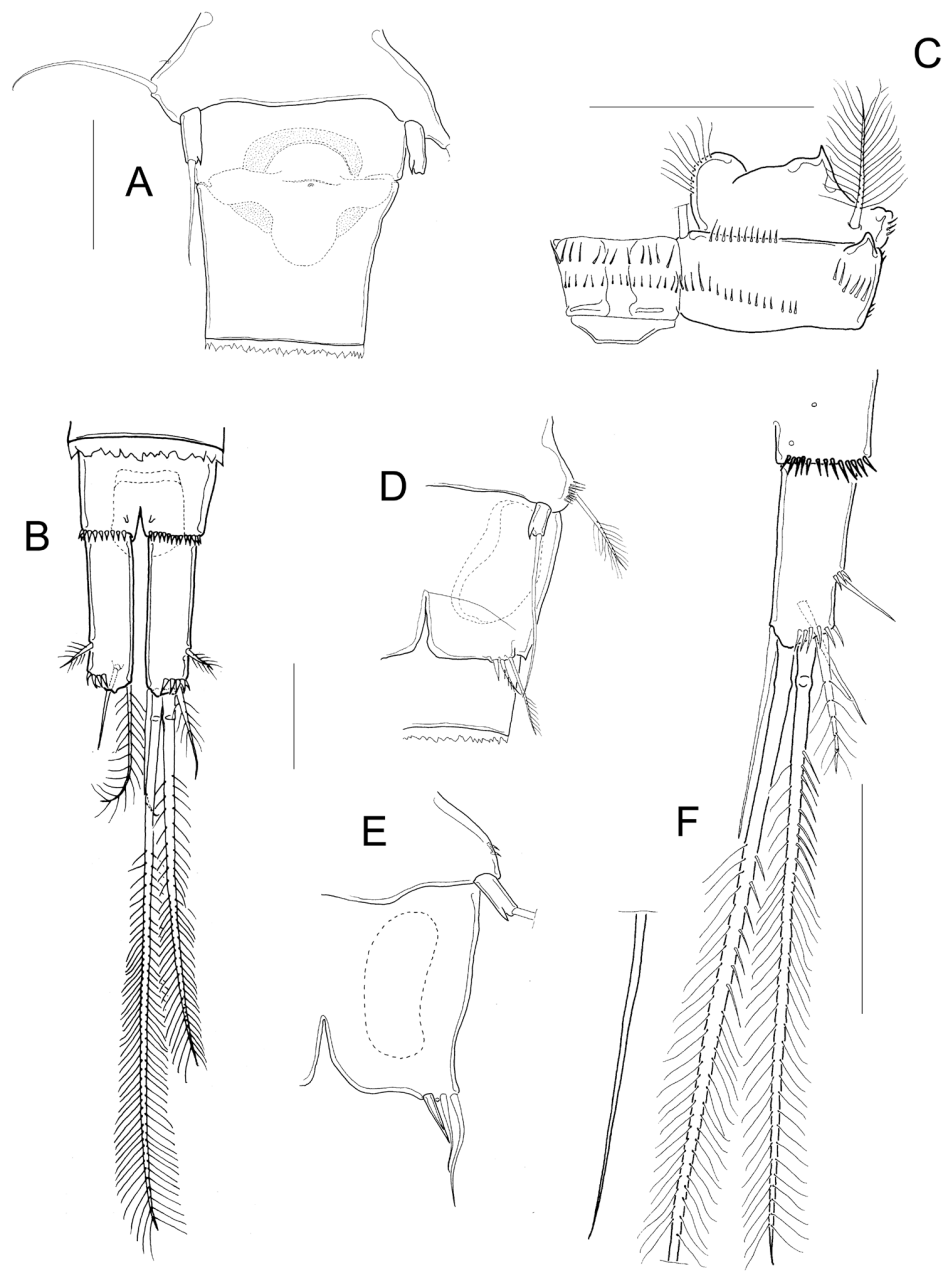
*Microcyclops anceps anceps* (Richard, 1897). Adult female. **A** Fifth pediger, genital double-somite (SMNK-2833) **B** Anal somite, caudal rami and caudal setae (Matillas), ventral. *Microcyclops anceps anceps*. Adult male. **C** P4 coxa, basis, and intercoxal sclerite, caudal (MNHN-Cp6877) **D** Fifth pediger and genital somite (MNHN-Cp6877) **E** Fifth pediger and genital somite (MNHN-Cp7295) **F** Anal somite, caudal rami and caudal setae (MNHN-Cp6876). Scale bars: 50 µm.

Figures 15 , 16 , 17

##### Description of female.

Dorsal posterior margin of second prosomal somite with crenulated hyaline fringe (Fig. 15A, B), posterior margin of fourth prosomal somite wrinkled (Fig. 15B). Caudal surface of antennal basis with three oblique rows of tiny spinules near inner margin and two basal (proximal) rows of long spinules near outer margin (Fig. 15C, D, F). Antenna with three-segmented endopod bearing 1, 9, and 7 setae, respectively. Frontal surface of antennal basis with three rows of spinules: one proximal oblique, one near lateral (outer) margin in middle of segment, and one next to exopod seta (Fig. 15E). Mandible with nine teeth on gnathobase (Fig. 15G). Maxillular palp with two armed and one naked setae apically; one armed seta plus two nude setae on lateral lobe, proximal seta with tiny spinules (Fig. 15H). Distal coxal endite of maxilla with two long setae: proximal seta with two long, basal setules and bifurcated apically; distal seta armed with a continuous row of tiny spinules along one (inner) margin (Fig. 15I). Basipodite with claw-like projection bearing two stout teeth followed by a row of tiny spinules, and one long smooth seta on its base; two-segmented Enp bearing 2 and 3 setae, respectively (Fig. 15I). Maxilliped with a row of spinules in syncoxa, Bsp, and Enp1, on frontal view (Fig. 15J).

Inner margin of *basipodite* with long and fine hairs in P1−P3 (Fig. 16A, B). One pore present on lateral margin of Enp2P1. Spine absent on inner margin of BspP1 (Fig. 16A). Inner margin of BspP4 with long. Inner margin of BspP4 with long spinules (Fig. 16D). Intercoxal sclerites naked in P1 and P2 (Fig. 16A, B). Usually one distal row or sometimes two rows of spinules present on intercoxal sclerite of P3 (Fig. 16C). P4 sclerite with two rows of spinules (Fig. 16D): spinules in distal row larger and stronger than those in proximal row (Fig. 16D). Medial apical spine of Enp2P4 1.3 ± 0.1 times as long as lateral apical spine, and 0.7 ± 0.03 times as long as segment; length to width ratio of segment 2.5 ± 0.1.

Strong spinules present (Figs 16E, 17D) or absent (Fig. 17A) on *fifth pediger* near base of lateral seta of P5. This character shows both inter- and intrapopulation variation; in one population, the females do not have spinules next to lateral seta (MNHN-Cp7296, unfigured here), while the males of the same population do (Fig. 17E).

Free segment of *fifth leg* 2.5 ± 0.2 times longer than wide, with relatively large spinule in distal position (Figs 16E, 17A); free segment 0.4 ± 0.08 times as long as apical seta. Distal margin of anal somite with continuous row of strong spinules on ventral and dorsal surfaces (Figs 17B, F). Caudal ramus 3.7 ± 0.3 times longer than wide, inner margin naked. Spinules present at base of caudal seta III. Caudal seta II inserted at 71.1 ± 1.15% of caudal ramus length (Fig. 17B).

*Seta* VII and VI 0.5 ± 0.1 and 0.8 ± 0.1times as long as caudal ramus, respectively. Relative lengths of terminal caudal seta from outermost to innermost, 1.0 : 4.9 : 7.1 : 1.3 (Fig. 17B). Caudal setae IV and V with homonomous setulation, with hair-like setules only (Fig. 17B).

## Discussion

*Microcyclops anceps anceps* showed the least variation in the qualitative and morphometric characters even though specimens were examined from a wide latitudinal range (Venezuela, Mexico, Guyana, Brazil, Guatemala, and Uruguay).

*Microcyclops echinatus* (from southeastern Mexico) and *Microcyclops ceibaensis* (from Honduras and southeastern Mexico) appeared morphologically similar. Similarities between these species are in: the length and width ratio of Enp2P4; the P4 sclerite with two rows of spines; the length ratio of the terminal caudal setae III and IV; the presence of spines at the insertion of setae II, and III; and the heteronomous ornamentation of the spine on the inner margin of Bsp P1. But the features that separate the specimens of these species were the insertion of the caudal seta II (69.5% in *Microcyclops ceibaensis* vs. 73.2% in *Microcyclops echinatus*); the length and width ratio of the caudal ramus is 3.6 in *Microcyclops ceibaensis* while 5.9 in *Microcyclops echinatus*; the presence of spines on the fifth pediger in *Microcyclops echinatus* vs. absence of these spines in *Microcyclops ceibaensis*; and the ornamentation of the inner basis of P4 with spine-like setae in *Microcyclops ceibaensis* vs. short setae plus hair-like setae in *Microcyclops echinatus*.

In 1935, Kiefer described two new species Cyclops (Microcyclops) diversus and Cyclops (Microcyclops) alius from Uruguay. The microscopic observations performed here, support the opinion of Reid (1986) on the synonymy of *Microcyclops ceibaensis* and *Microcyclops diversus*. The specimens labelled as *Microcyclops diversus* sp. n. share all the morphometric features of the type series of *Microcyclops ceibaensis*. Additionally, the structure of P1, P3, P4, and P5; the armament of the caudal surface of the antenna, the number of setae on each endopodal segment of the antenna, and the entire morphology of the urosome, and the caudal ramus in *Microcyclops diversus* are indistinguishable from the states found in *Microcyclops ceibaensis*.

The type specimens labelled as *Microcyclops dubitabilis* (from Trou Caiman, Haiti) and *Microcyclops alius* (from Barra Sta. Luzia, Uruguay) were morphologically similar to: 1) specimens identified as *Microcyclops rubellus* [including the specimen analysed by Reid (1992)], 2) some specimens from Southeastern Mexico, 3) *Microcyclops alius* from Brazil described by Rocha (1998), and 4) another specimen labelled as *Microcyclops dubitabilis* from Guadeloupe. The micro-structural analysis showed that the following features are common in all of the above mentioned specimens: the number of setae on the endopodal segments of the antenna and the number of rows of spines on the caudal surface of antennal basis; the ornamentation of the setae of the maxillular palp; the shape of the maxilla, and in particular, the structure of the distal coxal endite, the basipodite, and the seta on the claw-like projection; the shape and length of the spine on the inner margin of P1 basis; the shape and ornamentation of the intercoxal sclerites and the inner basis of P1, and P4; the meristic characters of P4, all traits of the caudal rami setae; the structure of P5, the anal somite, and the caudal rami. Therefore, *Microcyclops alius* is considered here as a junior synonym of *Microcyclops dubitabilis*, as it was suggested by Rocha (1998). Also, several specimens recorded under the name *Microcyclops rubellus* in the Americas likely refer to *Microcyclops dubitabilis*.

*Microcyclops dubitabilis* clearly differs from *Microcyclops varicans* s. str. at least in the next features: the spines at the insertion of caudal seta III are present in *Microcyclops dubitabilis*, but absent in *Microcyclops varicans*; caudal ramus is 3.5-4 times as long as wide in *Microcyclops varicans* and shorter in *Microcyclops dubitabilis*. Medial spine of Enp2P4 is around 0.8 times as long as the segment in *Microcyclops dubitabilis*, whereas that in *Microcyclops varicans* is shorter (around 0.5); and the seta inserted at base of claw-like projection in the maxilla is armed only with strong teeth at its base in *Microcyclops varicans*, but this armament is more complex in *Microcyclops dubitabilis*. Therefore *Microcyclops dubitabilis* is not a synonym of *Microcyclops varicans*.

*Microcyclops rubellus* and *Microcyclops varicans* have been recorded in several regions of the world and were thought to be likely cosmopolitan (Reid 1992) and highly variable in morphology. This is especially the case for *Microcyclops varicans* (Franke 1989, Alekseev 2002). The type material of *Microcyclops rubellus* and *Microcyclops varicans* is probably lost and both species were originally described from North Europe (Sars 1863, Lilljeborg 1901). Our review of the descriptions and drawings of *Microcyclops rubellus* from some European localities however revealed differences between the European and American specimens here examined in the medial surface ornamentation of the basis of the fourth swimming leg, in the length proportion of the medial apical spine and the Enp2P4, and the ornamentation of the distal margin of the anal somite (see Einsle 1993). Hence, *Microcyclops rubellus* s. str. probably is not distributed in America.

### Remarks about the new species

The specimens from southeastern Mexico assigned to the new species *Microcyclops inarmatus* were morphologically similar to that from Laguna Rincon, Haiti identified as *Microcyclops dubitabilis* (SMNK-2391, 2392) and to the specimen examined by Reid (1992) and classified as *Microcyclops varicans*. The shared morphology of the antenna, maxilla, P1 to P5, and urosome is obvious in all of these specimens (figured and described in the descriptive section before).

*Microcyclops inarmatus* sp. n. can be distinguished from *Microcyclops varicans* by the following characters (see also Rylov 1948, Einsle 1993): *Microcyclops varicans* has a more elongated caudal rami (3.5-4 times as long as wide), the basipodite of P4 bears short spinules on inner margin, there are more setae on the second endopodite of A2, on the distal margin of anal somite the spinules are present ventral, lateral and dorsally; and the medial spine of the second endopod of fourth leg has around the half length of the segment. All these features clearly differ to *Microcyclops inarmatus* sp. n.

The analysis that included specimens from a wide latitudinal range showed a highly conserved morphology primarily in the inner region of each swimming leg and oral appendages. Thus, we may speculate that some reports of *Microcyclops varicans* and *Microcyclops rubellus* recorded in the Americas are in fact *Microcyclops inarmatus* sp. n. and *Microcyclops dubitabilis*, respectively.

*Microcyclops inarmatus* sp. n. has some similarities also to *Microcyclops dubitabilis*, but the following features differentiate these two species: setal formula of the antennal endopod (1, 9, 7 in *Microcyclops dubitabilis* vs. 1, 6, 7 in *inarmatus*); the ornamentation on the caudal surface of the antennal basis is less complex in *Microcyclops inarmatus* than in *Microcyclops dubitabilis*; setae on maxillular palp are more armed in *Microcyclops inarmatus* than in *Microcyclops dubitabilis*; and the basal seta inserted at base of claw-like projection in the maxilla is more simple in *Microcyclops inarmatus*, whereas *Microcyclops dubitabilis* has two opposite rows of different spines.

The inner margin of the basis of the first swimming leg has a long spine with heteronomous ornamentation in *Microcyclops inarmatus*, in comparison to the short, homonomously setulated spine on this site in *Microcyclops dubitabilis*. In addition, the inner margin of the basis of the fourth swimming leg bears long hair-like setae and the fourth sclerite is almost as long as wide in *Microcyclops inarmatus*, whereas in *Microcyclops dubitabilis*, this inner margin bears short setae and the fourth sclerite is wider than long. The free segment of the fifth leg has a tiny spine on the medial margin in *Microcyclops inarmatus* (not described in Reid (1992), but clearly observed in the slide USNM-251321), and this spine is absent in *Microcyclops dubitabilis*. Finally, the lateral caudal seta is located near the middle of the caudal ramus in *Microcyclops inarmatus*, whereas in *Microcyclops dubitabilis*, this seta is located in the distal third. In all of the material analysed, no spines were observed at insertion of outermost terminal caudal seta in *Microcyclops inarmatus*, whereas in *Microcyclops dubitabilis*, these spines were observed in every specimen.

Other species of *Microcyclops* which has 12-segmented antenna, caudal rami with innermost terminal caudal setae longer than outermost terminal caudal setae, spines present only ventrally on the distal margin of the anal somite, caudal rami short (no more than 3 times as long as wide), one spine on inner basis of P1, and the intercoxal sclerite of P4 quadrangular and naked, are *Microcyclops davidi* (Chappuis, 1922) and *Microcyclops richardi* (Lindberg, 1942). *Microcyclops inarmatus* sp. n. differs from these species in the surface ornamentation of P4 basipodite: long hair-like setules vs. short spine-like setules in *Microcyclops davidi* (*sensu* Mirabdullayev et al. 2002) and *Microcyclops richardi* (see Lindberg 1942). The genital double-somite in *Microcyclops inarmatus* is short around 0.8 times as long as wide – similar in *Microcyclops davidi* –, but it is elongated in *Microcyclops richardi*, around 1.4 times longer than wide; and the second endopodite of A2 bears 9 setae in *Microcyclops davidi*, but only 6 setae in *Microcyclops inarmatus*.

Additionally, in *Microcyclops richardi* the free segment of P5 has no spine on medial margin, and the medial spine of Enp2P4 is short (0.6 times the length of the segment) in comparison with the new species. Finally, the seta on the base of the claw-like projection of the maxillar basipodite, is armed with tiny spinules in *Microcyclops inarmatus*, whereas in *Microcyclops davidi* it bears strong teeth on its base.

### Remarks on *Microcyclops anceps pauxensis* Herbst, 1962 and Microcyclops anceps var. minor Dussart, 1984

*Microcyclops anceps pauxensis* and Microcyclops anceps var. minor, described from the Amazonian region and Venezuela respectively, are similar in the number of segments of A1 (12-segmented); the length ratio of Enp2P4 (2.35 vs. 2.46); the intercoxal sclerite of P4 with two rows of spines; the length ratio of the lateral and medial apical spines of Enp2P4 (0.64 vs. 0.51); the length ratio of the medial apical spine of En2P4 and the segment (0.7 in both species), the insertion of the caudal seta II (lateral) is at 68% of the caudal rami length in *Microcyclops anceps pauxensis*, and 70% in *Microcyclops anceps minor*, and the continuous row of spines along the ventral and dorsal margins of the anal somite.

However, according to Herbst (1962) and Dussart (1984), the inner basis of P1 is naked in *Microcyclops anceps pauxensis*, and hairy in *Microcyclops anceps minor*; the inner basis of P4 bears small setules in *Microcyclops anceps pauxensis* but this is naked in *Microcyclops anceps minor*. The ratio between the lengths of the caudal setae VI and III is lower in *Microcyclops anceps pauxensis* than in *Microcyclops anceps minor* (1.81 vs. 2.52); the ratio between the lengths of caudal seta VI and caudal rami is higher in *Microcyclops anceps pauxensis* (1.44) than in *Microcyclops anceps minor* (1.07); the length ratio between the free segment of P5 and distal seta of P5 is 0.18 in *Microcyclops anceps pauxensis*, and 0.34 in *Microcyclops anceps minor*. Other differences between both species were observed in the length ratio between the dorsal caudal seta and caudal ramus (1.5 in *Microcyclops anceps pauxensis* vs. 0.57 in *Microcyclops anceps minor*), and the spines on the base of the outer caudal seta (spinules present in *Microcyclops anceps pauxensis* vs. absent in *Microcyclops anceps minor*). All of these differences suggest that *Microcyclops anceps minor* is distinct from *Microcyclops anceps pauxensis*.

Therefore, these taxa may constitute different species. The evaluation of mouthparts and the ornamentation of the inner region of each swimming leg in the type material may facilitate species delimitation. Unfortunately, the type material of both “subspecies” was not available to us.

### Conservative characters among species

Based upon morphological and morphometric features, eleven species and two subspecies of *Microcyclops* recorded in America were recognized. The following set of characters distinguishes between species: the ornamentation of the caudal surface of the antennal basis; the ornamentation of the setae of the maxillular palp; the shape and armature of the distal coxal endite of maxilla; and the basal seta in front of the claw-like projection of the maxillar basis. Previously, similar structures have been useful for differentiating other Cyclopinae species, such as *Mesocyclops* (Van de Velde 1984a, 1984b, Hołyńska 2000).

Among the specimens examined, the organization of the spine pattern on the antennal basipodite is similar to that proposed by Van de Velde (1984b) for *Mesocyclops* which is more complex on the caudal side than on the frontal side. Additionally, the caudal surface ornamentation of the antennal basis in *Microcyclops* here examined is similar to that in most New World *Mesocyclops*: the simple ornamentation pattern found in Neotropical *Mesocyclops* was considered by Hołyńska (2000) and Wyngaard et al. (2010) as an ancestral state. The pattern observed in *Microcyclops* is much less complex in comparison to those reported for some eucyclopinae species from the genus *Macrocyclops* (Karanovic and Krajicek 2012), *Paracyclops* (Karaytug 1999) and *Eucyclops* (Alekseev et al. 2006, Mercado-Salas et al. 2015).

The micro-structures of the swimming legs as diagnostic characters have been explored in *Mesocyclops*. In *Mesocyclops*, the coxal and basis armament of the first and fourth trunk limbs are important (Van de Velde 1984a, 1984b). In *Eucyclops*, the coxal seta of P4 or the intercoxal sclerites of all trunk limbs are informative (Alekseev et al. 2006). Our results show that features such as the medial surface ornamentation of basis of all four legs, the shape and ornamentation of the sclerites of P1 to P4, the presence/absence or length and armature of the spine on the inner basis of P1, and the shape or armature of the free segment of P5 were useful for differentiating between species.

Important diagnostic morphometric features for *Microcyclops* were the relative position of the lateral seta on the caudal ramus; the relative length of the outermost terminal caudal seta (III) and the outer median terminal caudal seta (IV); the relative length of caudal seta III and the inner median terminal caudal seta (V); and the length: width ratio of caudal ramus. Traditionally, the length ratio of the second endopod and its apical spines of the fourth trunk limb have been used as features to separate species of *Microcyclops*; however, as in another genus such as *Eucyclops* or *Mesocyclops*, the surface micro-structures together with the integumental armature and the meristic characters of the caudal rami are more informative.

This study is the first attempt to clarify the taxonomy of the species of *Microcyclops* recorded in America using detailed morphological analysis.

## Conclusion

The microscopic analysis of oral and thoracic appendages facilitated better delineation of *Microcyclops* species recorded in America. The characters that better distinguish between species are the ornamentation of antennal basipodite, the armature of the coxal endite and basipodite of the maxilla, the surface ornamentation of the inner basis of P1, the structure of intercoxal sclerites of the trunk limbs, the length: width ratio of caudal ramus, the length proportion of the caudal setae, and the relative position of the lateral seta on the caudal ramus.

The analysis performed here show that *Microcyclops alius* is a junior synonym of *Microcyclops dubitabilis*, and support the opinion about the synonymy of *Microcyclops ceibaensis* and *Microcyclops diversus*.

*Microcyclops inarmatus* sp. n. can be distinguished from other known species of the genus by the unique combination of several characters such as: morphometric characters of the second endopodite of fourth trunk limb and caudal ramus, presence of 6 setae on the second endopodal segment of antenna, antennal basipodite with just one group of spinules on caudal surface, lack of ornaments on the intercoxal sclerites of all swimming appendages, absence of spinules at base of lateral caudal and outermost terminal caudal setae, and basipodites of first to fourth swimming legs with long hair-like setules on inner margin.

### Key to the American species of *Microcyclops* (females)

The key is mainly based on the analysis performed in the descriptive section of this manuscript. Original descriptions were consulted in those species in which no microscopic observations could be made [*Microcyclops anceps pauxensis* (Herbst 1962); Microcyclops anceps var. minor (Dussart 1984); *Microcyclops mediasetosus* (Dussart and Frutos 1985); *Microcyclops pumilis* (Pennak and Ward 1985); and *Microcyclops medius* (Dussart and Frutos 1986)].

**Table:** 

1	Cylindrical free segment of P5 smooth, without inner spine (Fig. 7I–K)	**2**
-–	Cylindrical free segment of P5 with inner spine (Figs 4G, H; 16E)	**5**
2	Base of the outermost caudal seta (III) with a row of spines (Fig. 8A–C)	**3**
–	Base of the outermost caudal seta (III) without a row of spines	**4**
3	Length (L): width (W) ratio of caudal ramus is 4.35; lateral caudal seta inserted at 69% of the total caudal ramus length; inner basis of P4 naked	***Microcyclops medius***
–	L: W ratio of caudal ramus is 2.48 ± 0.2; lateral caudal seta inserted at 71 ± 5.7 % of the total caudal ramus length (Fig. 8A–C); inner basis of P4 with short hair-like setae (Fig. 7E–H)	***Microcyclops dubitabilis***
4	L: W ratio of caudal ramus is 5 ± 1; lateral caudal seta inserted at 80 % of the total caudal ramus length	***Microcyclops furcatus***
–	L: W ratio of caudal ramus is 2.3 ± 0.6; lateral caudal seta inserted at 55 % of the total caudal ramus length	***Microcyclops pumilis***
5	Inner spine of the cylindrical free segment of P5 tiny, articulated, inserted medially, and does not reach the distal margin of the segment (Figs 4H, 11C, 13D)	**6**
–	Inner spine of the cylindrical free segment of P5 strong, unarticulated; inserted terminally, projected beyond the distal margin of the segment (Figs 16E, 17A)	**11**
6	Length ratio of the innermost (VI): outermost (III) caudal setae is 3.0; L: W ratio of caudal ramus is 2.3; lateral caudal seta inserted at 57 % of the total caudal ramus length	***Microcyclops mediasetosus***
–	Length ratio of the innermost (VI): outermost (III) caudal setae is 1.6 to 2.0; L: W ratio of caudal ramus is 2.7 to 6.0; lateral caudal seta inserted at 60 to 75 % of the total caudal ramus length	**7**
7	Inner basis of P1 with hair-like setae, medial spine absent (Fig. 14B); inner basis of P4 hairy (Fig. 14D); intercoxal sclerite of P1 nude; intercoxal sclerite of P4 armed	***Microcyclops finitimus***
–	P1 basis with medial spine (Figs 4A, B; 10B, C; 13A); inner basis of P4 hairy (Fig. 4F), or with strong spine-like setae (Figs 10I, K), or with a combination of both (Fig. 13C); intercoxal sclerite of P1 nude (Fig. 4A, B) or armed (Fig. 13A); intercoxal sclerite of P4 nude (Fig. 2D) or armed (Fig. 10K)	**8**
8	Inner basis of P1 naked, medial spine reaching the proximal half of Enp2P1 and with homonomous ornamentation; L: W ratio of caudal ramus is 5 to 6, with a row of spines at the base of the lateral caudal seta (II) that extends dorsally; and no spines at the base of the outermost caudal seta (III)	***Microcyclops elongatus***
–	Inner basis of P1 hairy, medial spine reaching the distal half of Enp2P1 and with heteronomous ornamentation (Figs 2C; 4A, B; 13A); L: W ratio of caudal ramus is 2.5 to 6, with or without spines at the base of both the lateral (II) and the outermost caudal seta (III)	**9**
9	Anal somite with a row of spines on ventral margin; no spines at the bases of the caudal setae II and III (Figs 5A, C–E); intercoxal sclerites of P1 to P4 unarmed (Fig. 4A–D); basipodite of P4 with long hair-like setae on inner margin (Fig. 2D); one group of spines on caudal view of antennal basis (Fig. 3B); L: W ratio of caudal ramus 2.5 ± 0.4	***Microcyclops inarmatus* sp. n.**
–	Anal somite with a row of spines along both ventral and dorsal margins; with spines at the bases of the caudal setae II and III (Fig. 11C); intercoxal sclerites of P1 to P4 armed (Fig. 10C–H, K); basipodite of P4 with strong spine-like setae (Figs 10I, K), or with a combination of hair-like setae and spinules (Fig. 13C); more than one group of spines on caudal view of antennal basis (Figs 9F; 12B); L: W ratio of caudal ramus between 3.2 to 6.3	**10**
10	Fifth pediger with spines on ventral and lateral surfaces (Fig. 13D); caudal ramus is 5.9 ± 0.4 times longer than wide (Fig. 13E); inner basis of P4 with heteronomous ornamentation: short spine-like plus long hair-like setae (Fig. 13C); caudal surface of antennal basis with two rows of long spines next to exopodal seta (Fig. 12B)	***Microcyclops echinatus***
–	Fifth pediger nude ventrally and laterally; caudal ramus is 3.6 ± 0.4 times longer than wide (Fig. 11C); inner basis of P4 with homonomous ornamentation: strong spine-like setae (Figs 10I, K); caudal surface of antennal basis without spines next to exopodal seta (Fig. 9F)	***Microcyclops ceibaensis***
11	No spines on the base of the caudal setae II and III; inner basis of P4 naked, unarmed	**Microcyclops anceps var. minor**
–	Spines on the base of caudal seta III, no spines on the base of the caudal seta II (Fig. 17B); inner basis of P4 ornamented (Fig. 16D)	**12**
12	W ratio of caudal ramus is 3.7 ± 0.3 (Fig. 17B); inner basis of P1 hairy (Fig. 16A); inner basis of P4 with long spine-like setae (Fig. 16D)	***Microcyclops anceps anceps***
–	L: W ratio of caudal ramus is 2.4; inner basis of P1 naked; inner basis of P4 with short hair-like setae	***Microcyclops anceps pauxensis***

## Supplementary Material

XML Treatment for Microcyclops inarmatus

XML Treatment for Microcyclops dubitabilis

XML Treatment for Microcyclops ceibaensis

XML Treatment for Microcyclops echinatus

XML Treatment for Microcyclops finitimus

XML Treatment for Microcyclops anceps anceps

## Supplementary materials

Supplementary material 1 Table 2 Data type: RTF file Explanation note: Biological material examined. This dataset is made available under the Open Database License (http://opendatacommons.org/licenses/odbl/1.0/). The Open Database License (ODbL) is a license agreement intended to allow users to freely share, modify, and use this Dataset while maintaining this same freedom for others, provided that the original source and author(s) are credited.Martha Angélica Gutiérrez-Aguirre, Adrián Cervantes-Martínez
