# Supplementary material for: Taxonomic evaluation of eleven species of Microcyclops Claus, 1893 (Copepoda, Cyclopoida) and description of Microcyclops inarmatus sp. n. from America
Source: Zookeys. 2016 Jul 6;(603):33–69. doi: 10.3897/zookeys.603.7480 (PMC4978000; doi:10.3897/zookeys.603.7480)
Supplement: Supplementary material 1 — Table 2 [file zookeys-603-033-s001.rtf]

Table 1. Biological material examined. 
Species	Slide/container number	
Microcyclops dubitabilis (Kiefer, 1934)	SMNK-2391, female, Laguna Rincon, Haiti. 1.02.1935	
	SMNK-2392, female, Laguna Rincon, Haiti. 1.02.1935	
	SMNK-2081, 3 females. Haiti, Trou Caiman (Type locality). 16.02.1933	
	MNHN-Cp5398, -5399, two females, Abd, + P5. Caracas, Venezuela. 8.01.1954	
	MNHN-Cp 6764, female, Pond with Characeae, Guadeloupe	
M. anceps anceps (Richard, 1897)	MNHN-Cp6876, female, Calabozo reservoir, Venezuela. 15.10.1981	
	MNHN-Cp6877, male, Calabozo reservoir, Venezuela. 15.10.1981	
	MNHN-Cp7296, female, Marais Pripriyiyi, Guyane, 23.X.1985	
	MNHN-Cp7295, male, Marais Pripriyiyi, Guyane, 23.X.1985	
	MNHN-Cp6918, female, São Carlos (SP), Brazil, 8.12.1982	
	MNHN-Cp7119, roadside ditch prés Taxisco, Guatemala, 26.2.1970	
	SMNK-2184, female, Abd + P5, Uruguay	
	SMNK-2832, female, A1-P4, Maria Farinka, Brasilien, Tümpel, 25.08.1935, Brazil	
	SMNK-2833, female, Abd + P5, Maria Farinka, Brasilien, Tümpel, 25.08.1935	
	SMNK-3099, 5w + 1m, A-P4, Laguna Larga Magallanes, Chile, 03.02.1931	
	ECOCH-Z-00685, female, Laguna Matillas, Tabasco, Mexico, 12.I.1998	
	ECOCH-Z-00692, females (five in one vial), Charco 6 Jonuta-Villahermosa, 11.I. 1998	
	Female, Laguna Matillas, Tabasco, Mexico, 12.I.1998. Personal collection	
	ECOCH-Z-00692, female (in four slides + one vial with 7 females), km 45 Jonuta-Villahermosa, Tabasco, Mexico, 11.I.1998	
	Female, Laguna El Pajonal, Tabasco, Mexico, 12.I.1998. Personal collection	
	ECOCH-Z-00765, male, Laguna Espino, Tabasco, Mexico, 13.I.1998	
M. ceibaensis (Marsh, 1919)	USNM-51392, Holotype, labelled as Cyclops ceibaensis Marsh 1919, Honduras. Type Cat Mo. 51392. Note: 	
	USNM-222298, Paratype, labelled as C. cubanensis. Acc: 319629 (1 of 1)	
	USNM-222299, Paratype, labelled as Cyclops ceibaensis Marsh, 1919 La ceiba Pond Honduras Acc: 319629 (1 of 2), ant.antennule.abd.ft.	
	USNM-222299, Paratype Cyclops ceibaensis Marsh, 1919 La ceiba Pond Honduras Acc: 319629 (2 of 2), urosome, maxilla, P4	
	ECOCH-Z-01036, female, km 51 Villa hermosa-Frontera, lado 2, Tabasco, Mexico. 13.I.1998	
	Eight females, and one male (in one vial), km 51 Villahermosa-Frontera, lado 2, Tabasco, Mexico. 13.I.1998. Personal collection	
	Female, km 51 Villahermosa-Frontera, lado 1, Tabasco, Mexico. 13.I.1998. Personal collection	
	Female, km 51 Villahermosa-Frontera, lado 1, Tabasco, Mexico. 13.I.1998. Personal collection	
	Female, km 51 Villahermosa-Frontera, lado 2, Tabasco, Mexico. 13.I.1998. Personal collection	
	Female, km 154 Benemérito-Palenque, Chiapas, Mexico. 17.IV.2000. Personal collection	
	Female, km 154 Benemérito-Palenque, Chiapas, Mexico. 17.IV.2000. Personal collection	
M. rubellus (Lilljeborg, 1901)
	USNM-251322, one dissected female mounted on slide and eight adult females, Florida; Everglades, Shark River, 1986. Acc: 372909, Col. R. Conrow, Det. J. Reid	
	Female, Dzonot, Quintana Roo, Mexico, 28.V.1999. Personal collection	
	Female, km 109-108 Flor de Cacao-Benemérito, Chiapas, Mexico. 16.IV.2000. Personal collection	
	Female (two slides), Km 154 Benemérito-Palenque, Chiapas, Mexico. 17.IV.2000. Personal collection	
	Female (in three slides), km 51 Villahermosa-Frontera, lado 2, Tabasco, Mexico. 13.I.1998. Personal collection	
	ECOCH-Z-0737, three females (in one vial) Charco desv. Balancán I, Tabasco, Mexico 30.I.1999	
	ECOCH-Z-0747, five females, one male (in one vial), Laguna 3 brazos, canal Tabasco, Mexico, 13.I.1998. 	
	ECOCH-Z-0754, two females (in one vial) Laguna 3 brazos, litoral Tabasco, Mexico. 13.I.1998	
	ECOCH-Z-0765, one female (in one vial), Laguna Espino, Tabasco, Mex, 13.I.1998	
	ECOCH-Z-0769, 16 females, one male (in one vial), km 20 Jonuta-Villahermosa, Tabasco, Mex, 13.I.1998	
	ECOCH-Z-0708, three females (in one vial), Laguna Lehugal, Tabasco, Mexico, litoral, 31.I.1999	
	ECOCH-Z-0716, 14 females (in one vial), Laguna Leona Vicario II, litoral 1 y 2, Tabasco, Mexico, 31.I.1999	
	ECOCH-Z-0723, 10 females (in one vial), Laguna Leona Vicario II, litoral 2, Tabasco, Mexico, 31.I.1999	
	Females (15 in one vial), Benemérito-Palenque, km 154 Chiapas, 17.IV.2000. Personal collection	
M. varicans (G. O. Sars, 1863)
	USNM-251321, Sites 6, 23, Shark river slough, Everglades National Park, Florida, USA Acc: 372909 (slide 2 of 7). 1986	
	USNM, Site 23, Shark river slough, Everglades National Park, Florida, USA Acc: 372909 (41 females, five males in one vial). 1986	
M. inarmatus n. sp. 	ECOCH-Z-0679, 7 females (in one vial), km 51 Villahermosa-Frontera, Tabasco lado 2, 13.I.1998	
	One female, Laguna El Pajonal, Tabasco, Mexico. 12.I.1998. Personal collection	
	Female (in two slides), km 51 Villahermosa-Frontera, Tabasco lado 1, 13.I.1998. Holotype (in two slides ECOCH-Z-09337)	
	10 females (in one vial), km 51 Villahermosa-Frontera, Tabasco lado 1, 13.I.1998. Paratypes (ECOCH-Z-09338)	
	One female Charco desv. Balancán I, Tabasco, Mexico, 30.I.1999. Personal collection	
M. echinatus (Fiers et al., 2000)	ECOCH-Z-01038, female, km 51 Villahermosa-Frontera, Tabasco, lado 2, 13.I.1998	
	Female, El Guanal, Litoral II, Tabasco, Mexico, 31.I.1999. Personal collection	
	Female (in three slides), km 51 Villahermosa-Frontera, Tabasco lado 1, 13.I.1998. Personal collection	
	Two females and two males (in one vial) Charco salida Comitán-Montebello, Chiapas, 14.IV.2000. Personal collection.	
M. alius (Kiefer, 1935)
Actually junior synonym of M. dubitabilis	SMNK-2204, Typus, female, Barra Sta. Luzia, Uruguay; G. Teich, 07.01.1933	
	SMNK-2189, female labelled as Microcyclops alius n. sp., Uruguay	
M. diversus (Kiefer, 1935)	SMNK-2188, 2 females, Uruguay, A1-P4, 19.11.1934	
	SMNK-2190, 2 females, Abd + P5, Uruguay, 19.11.1934	
M. finitimus (Dussart, 1984)	MNHN-Cp7294, female, Rorota, near Cayenne, French Guiana, 21.X.1985	
